# Proteome-wide mapping of immune features onto *Plasmodium* protein three-dimensional structures

**DOI:** 10.1038/s41598-018-22592-3

**Published:** 2018-03-12

**Authors:** Andrew J. Guy, Vashti Irani, James G. Beeson, Benjamin Webb, Andrej Sali, Jack S. Richards, Paul A. Ramsland

**Affiliations:** 1 Life Sciences, Burnet Institute, Melbourne, Australia; 2 Department of Immunology, Monash University, Melbourne, Australia; 3 Department of Medicine, University of Melbourne, Melbourne, Australia; 4 Department of Microbiology, Monash University, Clayton, Victoria, Australia; 5 University of California, San Francisco, San Francisco, California USA; 6 Department of Infectious Diseases, Monash University, Melbourne, Australia; 7 Victorian Infectious Diseases Service, Royal Melbourne Hospital, Melbourne, Australia; 8 School of Science, RMIT University, Bundoora, Australia; 9 Department of Surgery Austin Health, University of Melbourne, Heidelberg, Australia

## Abstract

Humoral immune responses against the malaria parasite are an important component of a protective immune response. Antibodies are often directed towards conformational epitopes, and the native structure of the antigenic region is usually critical for antibody recognition. We examined the structural features of various *Plasmodium* antigens that may impact on epitope location, by performing a comprehensive analysis of known and modelled structures from *P. falciparum*. Examining the location of known polymorphisms over all available structures, we observed a strong propensity for polymorphic residues to be exposed on the surface and to occur in particular secondary structure segments such as hydrogen-bonded turns. We also utilised established prediction algorithms for B-cell epitopes and MHC class II binding peptides, examining predicted epitopes in relation to known polymorphic sites within structured regions. Finally, we used the available structures to examine polymorphic hotspots and Tajima’s D values using a spatial averaging approach. We identified a region of *Pf*AMA1 involving both domains II and III under a high degree of balancing selection relative to the rest of the protein. In summary, we developed general methods for examining how sequence-based features relate to one another in three-dimensional space and applied these methods to key *P. falciparum* antigens.

## Introduction

Malaria is an infectious mosquito-borne disease caused by *Plasmodium* species, responsible for an estimated 429,000 deaths in 2015^1^. *P. falciparum* is the major cause of malaria-related mortality in humans, with *P. vivax* also contributing significantly to morbidity. A more complete description of the determinants for effective immune responses against the parasite is a crucial step in the development of a highly efficacious malaria vaccine. The structural state of an antigen is an important factor that contributes to selection of immunodominant epitopes^2^. Protein conformational states spans a continuum between rigid, well-defined 3-dimensional (3D) structures and completely disordered states^3–5^. Previously, we have explored the role that intrinsically disordered proteins play as potential antigens within *Plasmodium* species, with disordered domains displaying marked differences to structured domains including containing a paucity of MHC binding peptides, an increased number of tandem repeat segments and an increased proportion of polymorphisms^6^. In this study, we turn our attention to epitope location within structured protein domains. In particular, we utilise established B-cell epitope predictors and predictors of MHC binding, examining these features in relation to the location of immunologically relevant polymorphisms over regions of experimentally determined or modelled structure. Additionally, we incorporate structural information into a test for balancing selection, allowing for more powerful identification of structured regions under immune selection pressure.

Immunity against clinical malaria develops naturally following repeated exposure, with antibodies known to play a key role in this process^7,8^. Within a naturally exposed population, immune selection pressure on the malaria parasite helps drive the occurrence of high-frequency polymorphisms on key malaria antigens. The development of a humoral immune response requires recognition of antigen in its native state. As a result, antigen structure plays a large role in the determination of epitopes for a humoral immune response. In other words, immune selection pressure driven by antibody-antigen interactions also occurs at the level of three-dimensional (3D) protein structure. Thus, examination of polymorphic regions in the context of protein 3D structure may help illuminate particular structural regions that are important targets of natural immunity. A number of studies have explored the relationship between protein structure and immune responses within *Plasmodium* species, including work on AMA1 from various species^9–13^, CSP^14–16^, EBA-175^17^, MSPDBL2^18^ and MSP2^19^. The majority of these studies have examined the location of polymorphic residues on a protein structure for single antigens, which likely arise as the result of immune selection pressure on particular epitopes. Polymorphisms can also arise as a result of T-cell driven selection pressure, as has been described for key T-cell epitopes within the C-terminal domain of CSP^20,21^. Other tests of immune selection pressure include Tajima’s D, which can help identify departure from a neutral model of selection^22^. A number of studies have examined *Plasmodium* proteins under immune selection pressure (balancing selection) using a sliding window approach^9,10,23–26^, although all of these studies examine Tajima’s D in the context of the linear sequence and do not consider the spatial proximity of residues (i.e., residues that are distant in the linear sequence may be proximal in the 3D structure). Here, we incorporate residue spatial information into measures of immune pressure, using both known and modelled protein structures. We demonstrate that the consideration of protein structural information can give extra insights into the regions of a protein under immune selection pressure.

In summary, we show that polymorphic residues within *P. falciparum* are usually surface exposed and are enriched within secondary structure turn elements. Predicted B-cell epitopes are also typically located on highly surface exposed regions. In contrast, predicted MHC class II binding peptides are generally buried within the core of a protein, and do not seem to overlap with polymorphic residues to a significant extent, which suggests that high frequency polymorphisms are more likely driven by humoral immune responses rather than cellular immunity. Antibodies often recognise discontinuous epitopes, therefore it is important to consider the spatial arrangement of residues when examining antigenicity. Accordingly, we incorporate structural information into a modified Tajima’s D test, and assessed two polymorphic vaccine candidates, EBA-175 and AMA1. We identified strong signatures of balancing selection for a discontinuous region of *Pf*AMA1 bordering domains II & III.

## Methods

### Data sources

Protein sequences for *Plasmodium* species were obtained from PlasmoDB, v28 (www.plasmoDB.org)^27^. Plasmodium genomes used were *P. falciparum* 3D7, *P. knowlesi* Strain H, *P. yoelii* 17X, *P. chabaudi* chabaudi, *P. vivax* Sal-1, *P. berghei* ANKA and *P. reichenowi* CDC. Coordinates for experimentally determined structures were obtained from the Protein Data Bank (PDB) from the Research Collaboratory for Structural Bioinformatics (RCSB) website (www.rcsb.org)^28^, accessed on April 20, 2017. Data on polymorphisms from 65 Gambian isolates were obtained from PlasmoDB^24^.

### Identification of matching PDB structures

For each *Plasmodium* species examined, matching PDB structures were identified using a BLAST search against the PDB database, with an e-value cut-off of 10.0. A sequence identity threshold >90% was used, normalized to the length of the shorter sequence in the comparison. The NCBI-blast+ 2.3.0 package was used for all BLAST searches^29^. Redundancy in PDB structures was removed using a sequence identity cut-off of 90% to group similar structures using precomputed sequence similarity clusters available on the RCSB PDB database (http://www.rcsb.org/pdb/).

### Python BioStructMap package - Spatial averaging of data over a protein structure

The BioStructMap Python package was developed to map various features on a protein structure. BioStructMap contains methods that take a PDB file as input, alongside a set of data that is aligned to a reference sequence. The output is a map of this data on a three-dimensional (3D) structure using either a predefined or user-defined function, either with or without some level of spatial averaging. The package makes use of the BioPython *Bio.PDB* module for PDB file parsing and manipulation^30^ and the DendroPy package for calculation of Tajima’s D^31^. The source code is available at https://github.com/andrewguy/biostructmap. For all structural mapping work using BioStructMap, protein chains were treated as monomers (i.e. interactions between chains in multimeric complexes were ignored).

### Comparative structure modelling of P. falciparum structures

Template-based models of *P. falciparum* structures were created using ModPipe^32^, an automated software pipeline that utilises MODELLER for the generation of comparative protein structure models^33^. Models are accessible *via* ModBase (https://modbase.compbio.ucsf.edu/)^34^. *P. falciparum* 3D7 sequences were used to compute all models. Structural models were deemed to be reliable if they had a ModPipe Quality Score (MPQS) greater than 1.1. The MPQS accounts for sequence coverage, sequence identity, gaps in the alignment, the compactness of the model and various statistical potential Z-scores^34^.

### Calculation of Tajima’s D

Tajima’s D is a statistical test used to identify regions of sequence evolving under non-neutral selection^22^. Tajima’s D was used here to identify regions of sequence subject to balancing selection, in which a higher level of sequence diversity is maintained within a population than would be expected under a neutral model of selection. Balancing selection can arise as a result of immune selection pressure within a population, and is indicated by positive values of Tajima’s D. Tajima’s D was calculated using the DendroPy Python package, version 4.2.0^31^. For calculation of Tajima’s D using a standard sliding window approach, the protein coding region for each gene was selected based on the 3D7 reference strain, and the corresponding multiple sequence alignment used for a sliding window calculation of Tajima’s D. A window size of 102 base pairs (bp) and step size of 3 bp was used unless otherwise specified. For calculation of Tajima’s D with incorporation of protein structural information (referred to as spatially derived Tajima’s D), the relevant PDB file sequence was aligned to the 3D7 reference strain, and a radius of 15 Å used to extract surrounding residues for each central residue, with Tajima’s D calculated using codons for these surrounding residues. For a detailed description, see the BioStructMap documentation at https://github.com/andrewguy/biostructmap.

Sequences for calculation of Tajima’s D were generated using polymorphism data obtained from PlasmoDB. Polymorphic sites were included on the proviso that at least 50 of the 65 sequences had a reliable base call at that position (percentage isolates with a base call ≥ 76%), in line with the original study^24^. A read frequency threshold of 70% and a minimum read depth of 5 was used to identify reliable base calls.

### Calculation of relative solvent accessibility and secondary structure

Relative solvent accessibility (RSA) was calculated using the DSSP program (accessed via BioPython)^35^, using the maximum accessible surface area (ASA) values from Rost & Sander^36^. When considering a two-state definition of solvent accessibility, an RSA threshold of 20% was used, similar to approaches taken in previous studies^37^. Note that this threshold is very close to the median RSA value for the proteins considered in this study, effectively splitting the dataset in two. Secondary structure assignment was also performed using the DSSP program using the eight DSSP secondary structure classes^35^: H, Alpha helix; B, Beta bridge; E, Strand; G, 3-Turn Helix; I, 5-Turn Helix; T, Turn; S, Bend; –, Other/Coil.

### Amino acid propensity scales

Average hydrophilicity and hydrophobicity within a given radius were calculated using the Hopp-Woods hydrophilicity scale^38^ and the Kyte-Doolittle hydrophobicity scale^39^, respectively.

### Prediction of B-cell epitopes

Potential B-cell epitopes were assessed using both BepiPred 1.0^40^ and BepiPred 2.0^41^, as we have previously used BepiPred 1.0 when examining potential epitopes within disordered *P. falciparum* proteins^6^ but wished to utilise the more accurate BepiPred 2.0 algorithm. BepiPred 2.0 uses a linear protein sequence to predict conformational B-cell epitopes, and is trained on epitope data from experimental crystal structures.

### Analysis of MHC binding peptides

Prediction of peptide binding to MHC was performed with NetMHCII 2.2^42,43^, with a 15 residue peptide length and default settings (threshold = −99.9, P1 amino acid residue preference turned off). Selection of MHC II alleles for analysis was performed according to known MHC haplotype frequencies from within a Gambian population^44^, obtained from www.allelefrequencies.net^45^.

### Prediction of membrane-proximal regions

To assess the potential impact of membrane proximity, transmembrane regions were predicted using TMHMM v2.0^46^. Known and predicted GPI-anchored proteins were obtained from Gilson *et al*.^47^, and the position of the GPI omega site within these proteins was predicted using PredGPI^48^. Structured regions were considered to be proximal to a transmembrane region or GPI anchor if they were within 10 residues of either.

### Visualisation of protein structures

Protein structures and features mapped on them were visualised using PyMol^49^; feature values were saved into the B-factor column of a PDB file, and visualised using the *spectrum* command.

### Data analysis and statistics

The majority of data analysis was performed using the Anaconda distribution of Python 3.5. Data was stored in an SQLite database, and accessed using Python SQLAlchemy. Plotting was performed with the Python Matplotlib package, version 1.5.1^50^. Statistical analysis was performed using SciPy^51^.

## Results

### A Python package for spatial mapping of features onto PDB structures

**Figure 1:**
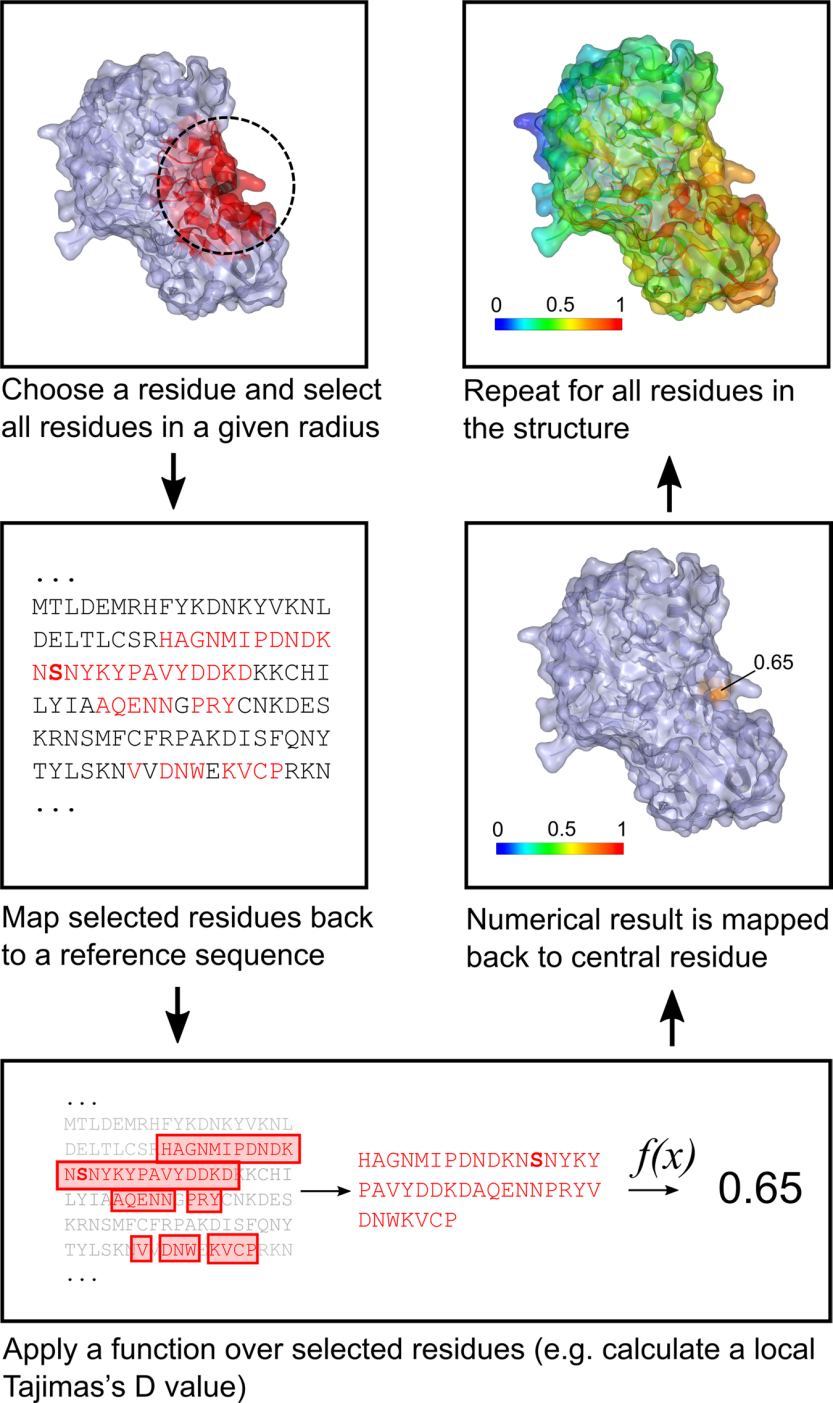
Overview of the structural mapping with spatial averaging approach as used in the Python BioStructMap package. For any given residue within a PDB structure, all residues within a specified radius of the given residues are identified. The location of these residues within a given reference sequence is then found, with the assumption that user-provided data will be aligned to this reference sequence. Using selected residues and the corresponding subset of user-provided data, a function is called, returning (usually) a numerical value. For example, this function may return the mean of the respective data. Note that the provided data and the mapping function may take a diverse number of forms. This includes functions which apply some statistical test over a multiple-sequence alignment of genetic sequences (e.g. Tajima’s D). In this case, the function would apply the statistical test over the subset of codons which code for the selected residues. The returned value is then assigned to the original residue in the PDB structure. This process is repeated for all residues within the PDB structure. Results can be viewed as a heatmap displayed over the PDB structure.

*Plasmodium* structures identified in the PDB were used for a series of spatial mapping analyses using our BioStructMap Python package. When dealing with protein sequence-level data, such as sequence polymorphisms or measures of evolutionary selection pressure, it is common practice to apply a function over a sliding window on the linear sequence. However, spatial proximity of residues within a 3D folded protein structure is often important, motivating an application of a “3D sliding window” across a protein structure as implemented in BioStructMap (Fig. 1). We were particularly interested in assessing immune-mediated selection pressure that occurs as a result of antibody recognition of a dominant epitope. Such selection pressure often applies to multiple residues within the epitope. Importantly, these residues are often not continuous within a linear sequence; therefore, consideration of protein structural information may enhance identification of regions of immunological importance. In this setting, the selection of a radius for calculation of adjacent residues should be reflective of the potential binding surface for an antibody. While the interaction dimensions for antibody-antigen interaction surfaces vary depending on the sequence of both antigen and antibody, the mean maximum paratope dimension were estimated as 28 Å^52^ or 30 Å^53^. We have used the upper of these two estimates when choosing a radius of 15 Å for all spatial averaging presented in this paper. Using data on sequence polymorphism from a study in The Gambia^24^, we mapped known polymorphisms onto *P. falciparum* crystal structures, correlating the occurrence of polymorphisms with residue surface exposure^36^, average hydrophilicity^38^ and hydrophobicity^39^, and predicted B-cell epitopes^41^ and MHC class II binding peptides^42,43^. We used the BioStructMap package to identify polymorphic hotspots within regions with known or modelled structure. Finally, we used this tool to include spatial information into a calculation of Tajima’s D and applied this to key antigens.

### Overview of experimental protein structures within Plasmodium species

To determine the number of proteins from various *Plasmodium* species that have structures in the PDB, we performed a BLAST search of proteins from major human, primate and murine *Plasmodium* spp. against a database of all PDB sequences. BLAST matches were grouped according to experimental technique (X-ray diffraction, NMR, other) and redundancy across PDB files was accounted for by clustering redundant PDB sequences using a 90% sequence identity threshold. For *Plasmodium falciparum* 3D7 sequences, there were a total of 368 non-redundant structures (total of 1,111 PDB files), equating to a total of 275 proteins (5.1% of the proteome) with at least one known structure in the PDB (Table 1). The majority of these structures were determined using X-ray crystallography. Similarly, for *P. vivax* Sal-1 sequences, 234 non-redundant structures (810 PDB files) were found, covering a total of 157 proteins (2.8% of the proteome).

**Table 1:** Number of proteins within various *Plasmodium* species which have at least one matching PDB structure, and number of unique PDB structures matched to various *Plasmodium* species grouped by experimental technique.

Species	Total protein coding genes	No. of proteins with PDB matches	Unique PDB Structures		
			All	X-ray Diffraction	NMR
*P. falciparum*	5398	275	368	229	31
*P. vivax*	5530	157	234	150	13
*P. knowlesi*	5316	124	176	99	10
*P. reichenowi*	5707	267	353	216	27
*P. berghei*	4952	108	143	81	8
*P. chabaudi*	5200	109	145	83	8
*P. yoelii*	5928	109	145	82	10

Note: Matching PDB structures were identified using a BLAST search against the PDB database, with an e-value cutoff of 10.0. A BLAST identity score of 90% was used as a cutoff for identifying close matches. When a PDB structure matched to multiple proteins within an organism, only the match with the highest identity score was considered for this table. Redundancy in PDB structures was removed using a Sequence Identity Cutoff of 90% to group similar structures using precomputed sequence identity clusters available on the RCSB PDB database (http://www.rcsb.org/pdb/). Plasmodium genomes used were *P. falciparum* 3D7, *P. knowlesi* Strain H, *P. yoelii* 17X, *P. chabaudi* chabaudi, *P. vivax* Sal-1, *P. berghei* ANKA, *P. reichenowi* CDC.

**Figure 2:**
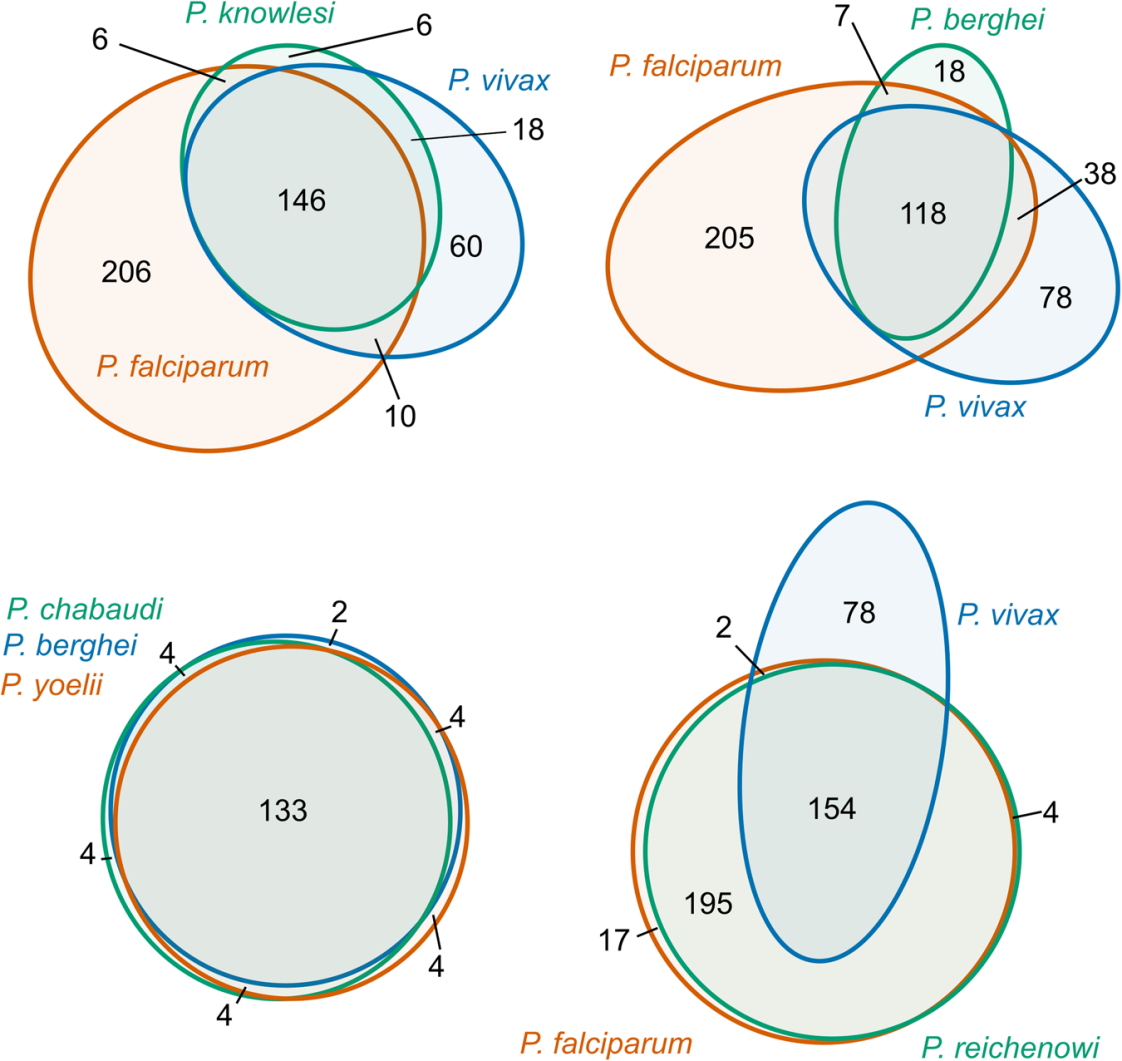
Comparison of unique protein structures which match to *Plasmodium* sequences with >90% identity. Euler diagrams show the number of PDB structures which match to single/multiple/all species. For clarity, only four representative combinations of species are shown. Matching PDB structures were identified using a BLAST search against the PDB database, with an e-value cutoff of 10.0. A BLAST identity score cutoff of 90% was used for included matches. Redundancy in PDB structures was removed using a Sequence Identity Cutoff of 90% to group similar structures using precomputed sequence identity clusters available on the RCSB PDB database (http://www.rcsb.org/pdb/). Only a single representative structure from each group of redundant structures was counted when generating Euler diagrams.

We examined the set of matching PDB structures for cross-species homology (Fig. 2). When comparing three human malaria species (*P. falciparum, P. vivax, P. knowlesi*), there was a large core of 146 non-redundant structures (596 PDB files) common to all 3 species, with another 206 non-redundant structures (490 PDB files) that matched only proteins in *P. falciparum*, while only 60 non-redundant structures (136 PDB files) matched proteins in *P. vivax* alone. For the 3 rodent malaria species examined (*P. yoelii, P. chabaudi, P. berghei*), 133 of the 155 non-redundant structures (602 of the 635 PDB files) matched proteins in all 3 species. Similarly, there was a large overlap between *P. falciparum* and *P. reichenowi*, with only 23 of the 372 non-redundant structures (58 of the 1127 total PDB files) matching proteins in only one of these two species. When considering all 7 species examined here, 81.2% (388 of 478) of non-redundant structures that matched proteins in any *Plasmodium* sequence also matched proteins in two or more *Plasmodium* species.

To determine the number of structured regions that were proximal to either transmembrane regions or GPI anchors, we predicted transmembrane regions using TMHMM v2.0^46^ and extracted known and predicted GPI-anchored proteins from Gilson *et al*.^47^. Only 5% (13 of 275) of proteins with known structures had structured regions within 10 residues of predicted transmembrane domains or GPI omega sites. Given the minimal number of membrane proximal structures identified, we have not treated membrane proximal regions differently in the following analysis.

### Polymorphic residues are predominantly surface-exposed

**Figure 3:**
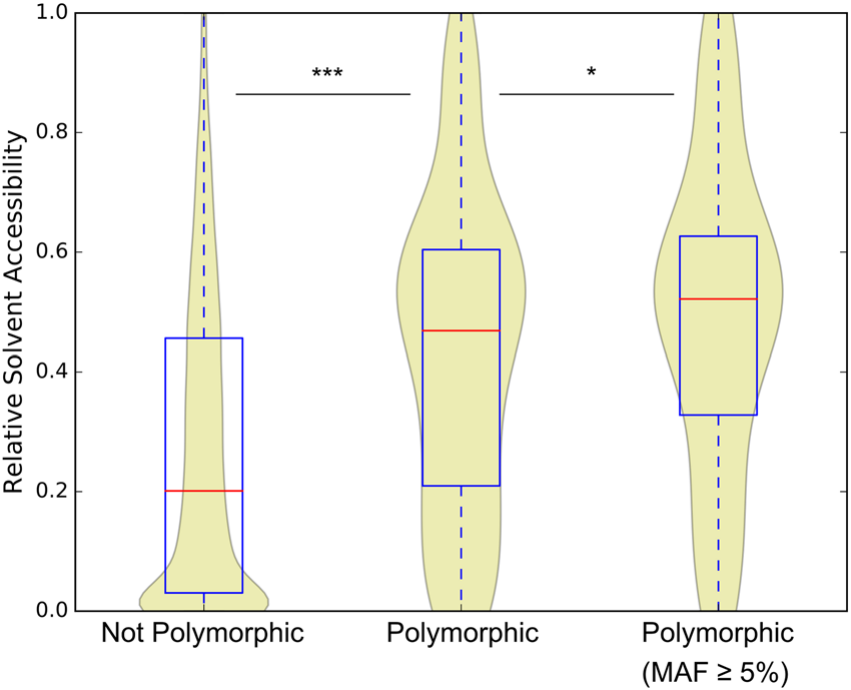
Polymorphic residues within known *P. falciparum* structures are predominantly surface exposed. Relative solvent accessibility (RSA) is shown for residues with and without identified polymorphisms. RSA represents the proportional surface area of a residue that is exposed to solvent, relative to the maximum possible exposure for that amino acid. RSA was calculated using the maximum accessible surface area (ASA) values from Rost & Sanders^36^. Box-and-whisker plots show median (red line) and interquartile range (box) of residue RSA values for each group. Violin plots show the smoothed distribution of RSA values for each group (violin plots employ a Kernel Density Estimation to compute an empirical probability distribution for each group). Polymorphic residues shown are both those with underlying non-synonymous SNPs regardless of allele frequency (n = 204), and those with underlying non-synonymous SNP with a minor allele frequency (MAF) ≥ 5% (n = 105). The majority of residues in the dataset did not have underlying polymorphisms (n = 28,869). Sequence polymorphisms were obtained from 65 Gambian isolates from Amambua-Ngwa *et al*.^24^, accessed via PlasmoDB. Polymorphic residues had significantly higher RSA values than the background RSA levels (p < 0.0001, Mann-Whitney U test), and polymorphic residues with a MAF ≥ 5% had significantly higher RSA than all polymorphic residues (p = 0.04, Mann-Whitney test).

We first correlated the occurrence of non-synonymous single nucleotide polymorphisms with the relative solvent accessibility (RSA) of the corresponding amino acid residues; RSA is a proportional measure of exposure of a particular amino acid residue to bulk solvent. Based on a few known examples^10,54^, it was expected that polymorphic residues that evolved to evade humoral immune responses would be located predominantly on the surface. We indeed observed that polymorphic residues generally had some level of solvent exposure, with higher overall RSA values than residues that contained no underlying nucleotide polymorphisms (Fig. 3). Polymorphic residues had a significantly higher median RSA value of 0.47 than the background median RSA level of 0.20 (p < 0.0001, Mann-Whitney U test). Residues with underlying synonymous SNPs did not have significantly different RSA values to the background distribution (p = 0.79, Mann-Whitney U test). Immunologically relevant polymorphisms are expected to be maintained at a high frequency within a population. A minor allele frequency (MAF) threshold of 5% is commonly used to distinguish between high- and low-frequency polymorphisms^55–58^. Correspondingly, polymorphic residues with a MAF ≥ 5% had significantly higher RSA than all polymorphic residues (p = 0.04, Mann-Whitney test), with a median RSA of 0.52.

### Average hydrophilicity and hydrophobicity in relation to polymorphic residues

A number of epitope prediction methods have utilised amino acid residue propensity scales based on residue biophysical properties such as hydrophobicity and hydrophilicity in an attempt to predict targets of adaptive immune responses^38,40,59–62^. We considered here the effect of average residue hydrophobicity and hydrophilicity within a 15 Å radius on polymorphism location, but observed only a small increase in hydrophilicity and corresponding small decrease in hydrophobicity (Figure S1a,c). Given that average hydrophilicity and hydrophobicity are correlated weakly with RSA, it is likely that the small shift in average hydrophilicity and hydrophobicity is reflective of the much larger shift observed for solvent accessibility between polymorphic and non-polymorphic residues. To explore this hypothesis, we re-ran the spatial averaging algorithm and restricted it to surface exposed residues (RSA ≥ 0.2), showing only a small, albeit significant, difference between polymorphic and non-polymorphic residues (Figure S1b,d).

### Polymorphic residues occur preferentially in turns

We also investigated whether or not polymorphic residues are more likely to occur in any particular secondary structure motifs. When considering all polymorphic residues, we observed a significant reduction in polymorphic residues within β-strand elements (E), from 19% to 11% (p = 0.006) (Figure S2). When considering only those polymorphisms with MAF ≥ 5%, we observed a large reduction in the proportion of residues within β-strand elements (E) (p = 0.0009), from 19% to 6%, alongside a large increase in the proportion of residues within turns (T), from 9% to 22% (p < 0.0001).

### Prediction of B-cell epitopes and MHC class II binding peptides

Polymorphisms that arise as a result of immune-mediated selection pressure may be driven by antibody or T-cell responses. T-cells recognise peptide antigen in the context of MHC molecules, whereas antibodies typically recognise antigen in its native state. In the context of a humoral immune response, CD4+ T-cell responses are important for the development of a T-dependent B-cell response, recognising peptide antigen presented by B-cells on MHC class II molecules. To examine the location of predicted B-cell epitopes in relation to regions of known structure in *P. falciparum* we used the newly released BepiPred 2.0^41^ (Figure S3). Although a number of tools exist for predicting B-cell epitopes based on structural data^63,64^, we wished to assess the utility of a state-of-the-art method that only uses the linear protein sequence as input, as this will be applicable to the many *Plasmodium* proteins with unknown structures. As we have previously utilised BepiPred 1.0 in a study examining disordered proteins in *P. falciparum*^6^, we provide a comparison to the older BepiPred 1.0^40^ algorithm here (Figure S4). Our subsequent analysis was only performed on proteins that contained at least one high frequency polymorphic residue within a region of known structure, as it was impractical to run the full proteome through the BepiPred 2.0 online interface. Epitopes predicted with BepiPred 2.0 were predominantly surface exposed, with increasing BepiPred thresholds predicting increasingly surface exposed residues (Figure S3a). BepiPred 2.0 epitope scores were significantly higher for polymorphic residues (p < 0.0001; Mann-Whitney U test), even when restricting analysis to surface exposed residues (RSA ≥ 0.2) (p < 0.0001; Mann-Whitney U test) (Figure S3b). At the default threshold, 50% of non-polymorphic surface-exposed residues fell within predicted epitopes, compared to 75% of polymorphic surface-exposed residues.

We also examined the location of predicted MHC class II binding peptides, specifically looking at MHC class II haplotypes, again using those that have been shown to be present at high frequency within the Gambian population: HLA-DPA1*02:01-DPB1*01:01 and HLA-DQA1*05:01-DQB1*03:01^44^. MHC class II binding peptides were observed to be predominantly buried within the core region of proteins, with lower overall surface exposure than the set of non-binders (Figure S3c, d). Additionally, polymorphic residues were found at lower frequency within MHC class II binding peptides compared to non-binding regions. For the HLA-DQA1*05:01-DQB1*03:01 haplotype, 0.20% of residues involved in high-binding peptides were polymorphic (MAF ≥ 5%), compared to 0.74% of residues not involved in a predicted MHC binding peptide. Similarly for the HLA-DPA1*02:01-DPB1*01:01 haplotype, 0.21% of residues involved in high-binding peptides were polymorphic (MAF ≥ 5%), compared to 0.71% of residues not involved in a predicted MHC binding peptide. Thus, MHC class II binding peptides are typically buried within the hydrophobic core of proteins, and do not appear to be significant targets of immune selection pressure.

### Comparative structural modelling of Plasmodium falciparum proteins

To extend the structural coverage, the entire proteome of the *P. falciparum* (3D7 strain) was subjected to comparative structural modelling using ModPipe^32^ (https://salilab.org/modpipe/) based on structures in the PDB. A MPQS threshold of 1.1 was used to filter out low-quality models before further analysis. A total of 1575 reliable models were created, covering 923 proteins or 17% of the proteome. The majority of the models in the filtered dataset covered the entire length of the corresponding *P. falciparum* 3D7 protein sequence (median coverage = 95%, mean coverage = 87%) (Figure S5). We then used both known and modelled structures to identify polymorphic hotspots within *P. falciparum*.

### Identification of polymorphic hotspots

**Figure 4:**
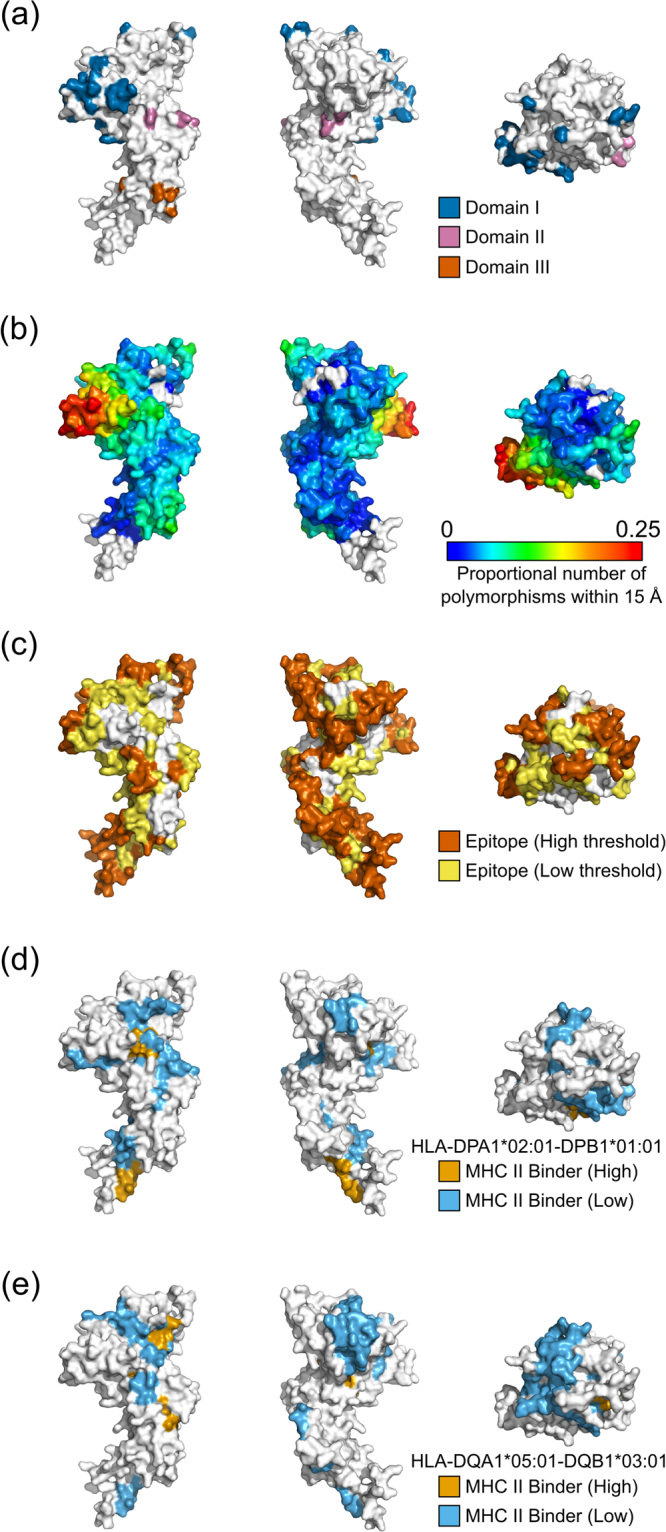
Location of immunologically relevant features mapped onto a *Pf*AMA1 structural model. Each panel shows the front, back and top view of the modelled *Pf*AMA1 structure. (**a**) Polymorphic residues with an underlying minor allele frequency (MAF) greater than 5% are shown colored according to location within domain I (blue), domain II (magenta) or domain III (orange). Sequence polymorphisms were obtained from 65 Gambian isolates^24^. (**b**) Spatial averaging of polymorphic residues highlights polymorphic hotspots. The proportion of polymorphic residues within 15 Å is shown for each central residue, with polymorphic residues defined as those with a MAF ≥ 5%. (**c**) Bepipred 2.0 predictions are shown over the *Pf*AMA1 structure, with epitopes shown for two Bepipred thresholds—predicted epitopes are shown in yellow for a threshold of 0.5 (specificity = 0.57, sensitivity = 0.59) and in dark orange for a threshold of 0.55 (specificity = 0.81, sensitivity = 0.29). (**d,e**) The location of predicted MHC class II binding peptides are shown for the HLA-DPA1*02:01-DPB1*01:01 (**d**) and HLA-DQA1*05:01-DQB1*03:01 (**e**) alleles. Residues involved in a low binding peptide (50 nM < IC50 < 500 nM) are shown in light blue, while residues involved in a high binding peptide (IC50 < 50 nM) residue are shown in orange. Only the core binding region of each peptide binder is indicated on each structure.

**Figure 5:**
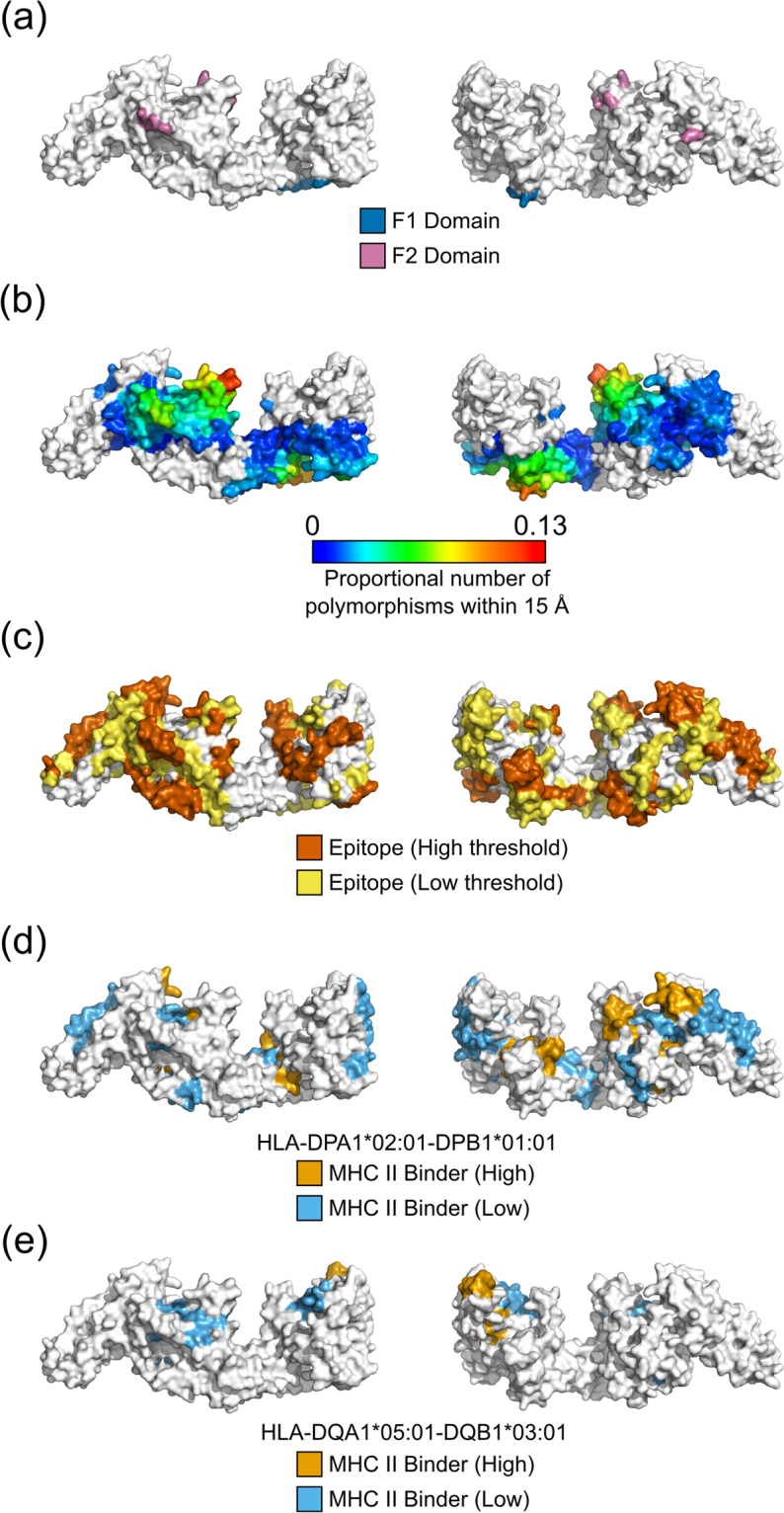
Location of immunologically relevant features mapped onto an EBA-175 RII homology model. Each panel shows the front and back view of the modelled EBA-175 structure. The homology model was modelled from the 1ZRO PDB structure using ModPipe. (**a**) Polymorphic residues with an underlying minor allele frequency (MAF) greater than 5% are shown colored according to location within Region I (blue) and Region II (magenta). Sequence polymorphisms were obtained from 65 Gambian isolates^24^. (**b**) Spatial averaging of polymorphic residues highlights polymorphic hotspots. The proportion of polymorphic residues within 15 Å is shown for each central residue, with polymorphic residues defined as those with a MAF ≥ 5%. (**c**) Bepipred 2.0 predictions are shown over the EBA-175 structure, with epitopes shown for two Bepipred thresholds: predicted epitopes are shown in yellow for a threshold of 0.5 (specificity = 0.57, sensitivity = 0.59) and in dark orange for a threshold of 0.55 (specificity = 0.81, sensitivity = 0.29). (**d,e**) The location of predicted MHC class II binding peptides are shown for the HLA-DPA1*02:01-DPB1*01:01 **(d)** and HLA-DQA1*05:01-DQB1*03:01 **(e)** alleles. Residues involved in a low binding peptide (50 nM < IC50 < 500 nM) are shown in light blue, while residues involved in a high binding peptide (IC50 < 50 nM) residue are shown in orange. Only the core binding region of each peptide binder is indicated on each structure.

Polymorphic regions have often been thought to relate to potential antigenicity of malaria proteins, with antigenic diversity a contributing factor to parasite evasion of host immune responses^65,66^. With this in mind, spatial averaging of polymorphisms was performed to identify regions of proteins that have clusters of high-frequency polymorphisms and are hence likely to be under some level of immune selection pressure. Given that polymorphic residues tend to be surface exposed, we have restricted this analysis to surface exposed residues with RSA ≥ 0.2. Polymorphic hotspots on *P. falciparum* structures were identified using the set of all PDB crystal structures that matched proteins in *P. falciparum* (Table S1), and the set of all modelled structures with MPQS > 1.1 (Table S2). Protein polymorphisms from the Gambian population were used, and a MAF threshold of 5% employed to restrict polymorphisms to those with some immunological relevance. A threshold of either 10% or 20% of surrounding residues being polymorphic was used to identify regions of protein structure that are particularly polymorphic. For both experimental and modelled sets of structures, most proteins identified are known antigens, and include AMA1, CSP, TRAP, PfEMP1, DBL-MSP2, MSP1 and EBA-175. Mapping of polymorphisms and predicted B-cell epitopes and MHC class II binding peptides on these structures is shown in Figures S6–S10 & Figs 4 and 5. We have used known structures where there are no missing residues, and modelled structures when the relevant experimental structures have unresolved residues. Although the density of polymorphic residues differs between antigens, all proteins examined here have regions of particularly dense polymorphisms that in most cases overlap with B-cell epitopes predicted using BepiPred 2.0. In many cases, the most polymorphic regions are surface exposed protrusions.

### Key parameters mapped to PfAMA1 & EBA-175

From the proteins identified above, we focus here on two major vaccine candidates for *P. falciparum* malaria: Apical Membrane Antigen 1 (AMA1) and Erythrocyte Binding Antigen 175 (EBA-175) Region II (RII) **(**Figs 4 and 5). A number of crystal structures exist for *Pf*AMA1, but they only encompass domains I and II. Previous studies suggest that domain III may also be a significant target of protective antibody responses^10,67^, and hence we used a modelled structure of all three domains to calculate polymorphic hotspots and spatially derived Tajima’s D values. There are two models for *Pf*AMA1 generated by ModPipe with MPQS > 1.1; one of these models uses a *Pf*AMA1 structure as a template and hence does not include domain III, whereas the other model uses a *Pv*AMA1 structure as a template and includes all three domains. However, this second model fails to accurately place a loop (S345-Y397; 3D7 sequence) that is unresolved within the *Pv*AMA1 template but resolved within known *Pf*AMA1 structures. Previously published work has manually modelled the full domains I-III of *Pf*AMA1 using a combination of *P. falciparum* and *P. vivax* templates, and we have used this model for examining *Pf*AMA1^10^. While the crystal structure for EBA-175 RII has been solved^68^, we have used a ModPipe homology model for analysis here as this model includes a number of residues that are unresolved in the experimental structure. These unresolved residues occur in loops that are no more than 4 residues long, and as such the model is likely not to be inaccurate.

For *Pf*AMA1, the majority of polymorphic residues are surface exposed, with most polymorphisms falling within Domain I (Fig. 4a). The most polymorphic region (Fig. 4b) is a highly surface-exposed loop formed by residues T194 - D212, which has been termed the C1-L cluster^69,70^. Potential B-cell epitopes were predicted using BepiPred 2.0 (Fig. 4c). The predicted epitopes (especially at higher thresholds) predominantly fell on highly surface-exposed regions. In contrast, predicted MHC class II binding peptides were predominantly buried within the core of the protein, especially for predicted high-affinity peptides (Fig. 4d,e). For MHC class II haplotypes with high frequency within the Gambian population (e.g., the HLA-DPA1*02:01-DPB1*01:01 haplotype), 50% of residues involved in predicted high-binding peptides were located in the core of the protein (RSA < 0.2), while 57% of residues involved in low-binding peptides were also buried. For HLA-DQA1*05:01-DQB1*03:01, 83% and 43% of residues from predicted high- and low-binding peptides, respectively, were buried in the protein core (47% of all residues are buried).

When considering EBA-175 RII polymorphisms (Fig. 5a), two main hotspots were identified, with both being surface-exposed loops (Fig. 5b). These two loops are located on opposing faces of the structure, with one loop within the F1 domain (residues N252–V266) and the other within the F2 domain (residues S432 - N442). The F2 surface loop is involved in the formation of a two-strand antiparallel β-sheet between identical residues (N433 - H436) upon EBA-175 RII dimer formation^68^. This region is in the center of the RII dimer, with residues K439 and K442 also likely involved in binding to glycans in the glycophorin A receptor. The F2 β-finger (residues C476–C488) that is the target of inhibitory monoclonal antibodies R217 and R215^17,71^ was also identified as a polymorphic hotspot, although to a lesser extent. Similar to *Pf*AMA1, predicted B-cell epitopes were located on highly surface-exposed regions of EBA-175 RII (Fig. 5c) and include the known F2 β-finger epitope and an epitope (H303 - Q315) that is the target of the R218 monoclonal antibody^72^. Conversely, MHC class II binding peptides were mostly buried (Fig. 5d,e). For the HLA-DPA1*02:01-DPB1*01:01 haplotype, 70% of residues involved in predicted high-binding peptides were located in the core of the protein (RSA < 0.2), while 58% of residues involved in low-binding peptides were also buried. For HLA-DQA1*05:01-DQB1*03:01, 50% and 65% of residues from predicted high- and low-binding peptides respectively were buried in the protein core (44% of all residues are buried). In summary, a number of highly polymorphic surface exposed regions were identified for both *Pf*AMA1 and EBA-175 RII, which in most cases overlapped with predicted B-cell epitopes. In contrast, predicted MHC II binding peptides were predominantly buried within the core of the protein.

### Incorporating protein spatial information into a genetic test for immune selection pressure

**Figure 6:**
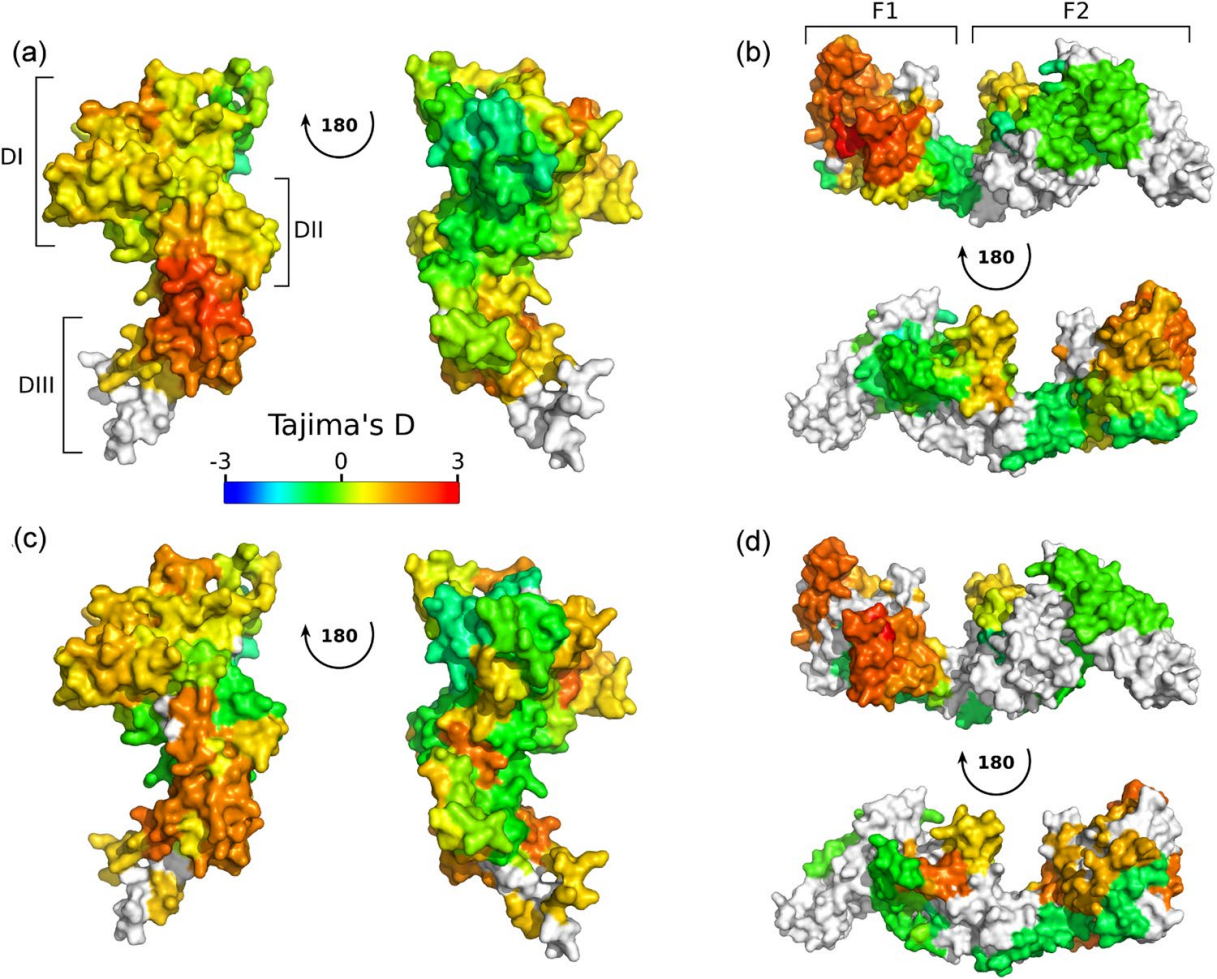
Calculation of Tajima’s D for *Pf*AMA1 and EBA-175, both with and without incorporation of protein structural information. (a, b) Spatial information incorporated into a calculation of Tajima’s D using modelled protein structures for AMA1 (**a**) and EBA-175 RII (**b**). Tajima’s D values for each residue were calculated using only those codons which were mapped to residues within a 15 Å radius of the central residue. (**c**,**d**) Tajima’s D was calculated over a sliding window of 102 bp and a step size of 3 bp, without incorporation of protein structural information. Tajima’s D values for central codons are displayed on the modelled protein structures for AMA1 (**c**) and EBA-175 RII (**d**). Data on sequence polymorphisms was obtained from PlasmoDB using sequences from 65 Gambian isolates^24^. The structural model for *Pf*AMA-1 was manually modelled, previously published in Arnott *et al*.^10^, and covers domains I-III of *Pf*AMA-1. The structural model for EBA175 RII was created using Modpipe, with the PDB structure 1ZRO used as a template. Structures are colored according to the calculated value of Tajima’s D mapped to each residue, with residues without a defined Tajima’s D value shown in white.

To assist in the identification of regions of protein under immune selection pressure, we developed a modified calculation of Tajima’s D that includes protein structural information using the spatial averaging approach introduced earlier in this study. We compared this approach to application of a standard sliding-window over the linear sequence to assess whether our spatially derived Tajima’s D calculation improved the ability to detect sites under immune selection pressure. These two methods were applied to *Pf*AMA1 and EBA-175 (Fig. 6). For AMA1, there is evidence for balancing selection within DI when calculating Tajima’s D using a traditional sliding window approach (Fig. 6c), as has been observed previously in other populations^10^. In contrast to the linear sliding-window approach, the new spatial averaging approach reveals a surface exposed region on the border of DII and DIII as the area with the highest Tajima’s D values; parts of DI also appear to be under balancing selection (Fig. 6a). As expected, the so-called ‘silent face’ of *Pf*AMA1 had Tajima’s D values that were negative or close to zero. For EBA-175, a large portion of the F1 domain appears to be under balancing selection, as is a surface loop in the F2 domain (residues S432 - N442) (Fig. 6b,d). The region with highest calculated spatially derived Tajima’s D values is contained within the F1 domain of RII, comprised predominantly of residues E266-D289, P314-Q322 and L382-L400. This site is also part of the dimerization interface formed between two molecules of EBA-175 RII as it binds to its glycophorin A receptor; during dimerization this site makes contact with the F2 domain of the other dimer pair^68^. It has previously been suggested that antibodies that block dimerization of EBA175 may negatively impact on glycophorin A engagement^71^, and antibodies that inhibit binding of EBA-175 to glycophorin A have also been shown to be associated with protection from clinical malaria^72^. Results from spatially derived Tajima’s D and conventional sliding window Tajima’s D analysis are similar to each other for EBA-175. This structure is predominantly alpha-helical, and sites with high Tajima’s D values are mostly continuous stretches of protein sequence.

**Figure 7:**
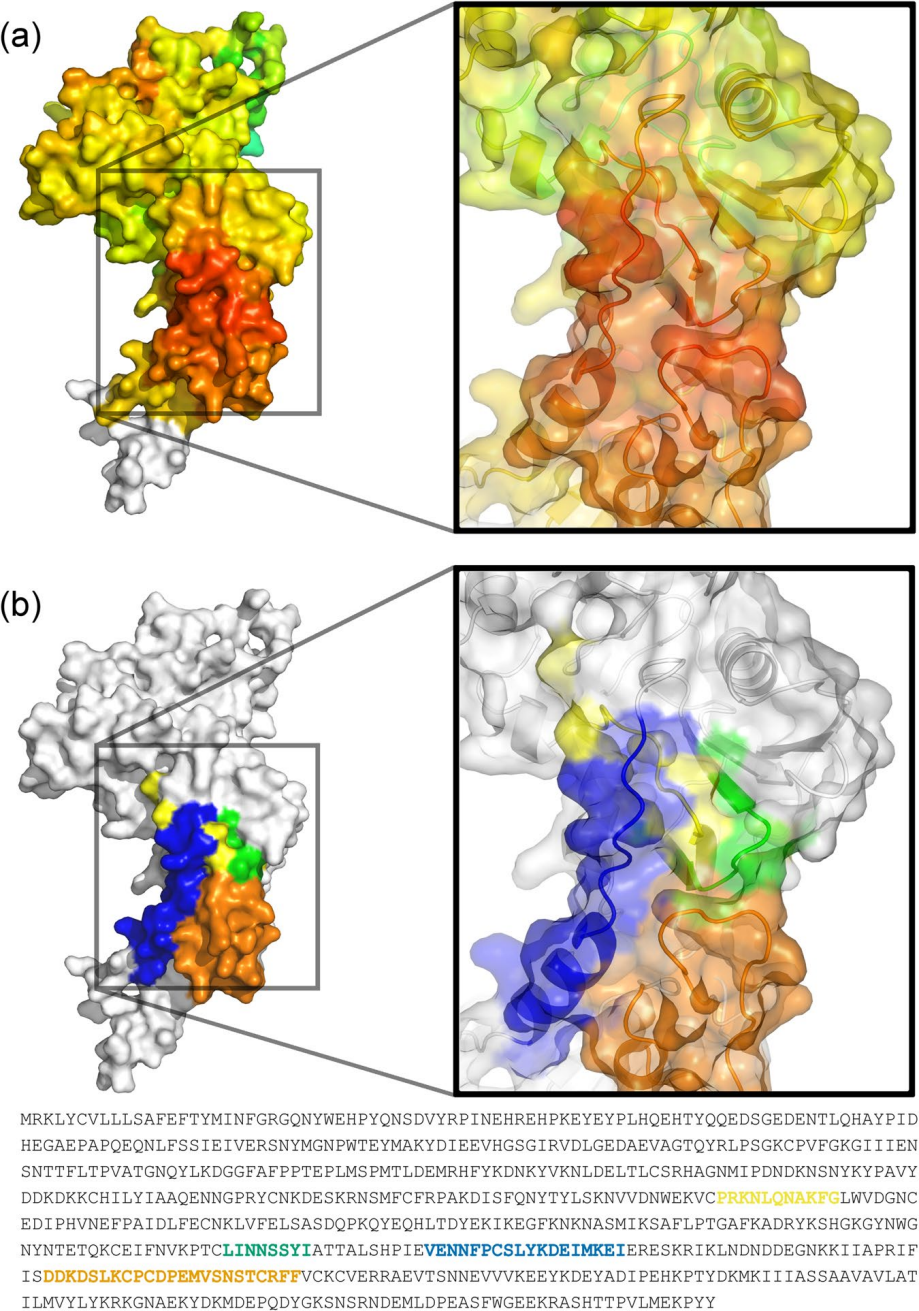
Four discontinuous stretches of sequence make up a region of *Pf*AMA1 with high Tajima’s D values as calculated using spatial mapping. (**a**) Detailed view of a region of *Pf*AMA1 with high Tajima’s D values as calculated using spatial averaging. The protein structure is colored according to the color scale presented in Fig. 6, with red residues corresponding to the highest Tajima’s D values. Without the use of spatial averaging, there is a maximum Tajima’s D value of 1.84 within this region, whereas a maximum value of 2.39 is observed when incorporating spatial information. (**b**) Four discontinuous regions of sequence contribute to the set of surface exposed residues with highest Tajima’s D values. These four regions are shown in yellow (P303 - G313; DII), green (L419 - I426; DII), blue (V437 - I454: DII/III) and orange (D483 - F505; DIII).

Previous analyses of the cross-reactivity of human antibodies, and vaccine-induced antibodies in rabbits, to different AMA1 alleles^73,74^ and mutation studies of C1 residues^75^ suggested that polymorphisms in the C1 cluster did not explain a large component of antigenic differences between alleles. Furthermore standard analysis of polymorphisms in linear sequences did not correlate highly with antigenic differences^74,75^. Therefore, additional approaches that consider structure may be required to yield further insights. Interestingly, our analysis of AMA1 incorporating structural considerations identified a site within DII/DIII of AMA1 that stands out as having a high spatially derived Tajima’s D score. This site is composed of four distinct regions of continuous sequence (P303 - G313; L419 - I426; V437 - I454; D483 - F505), which together make up a surface exposed face of DII/DIII (Fig. 7). The spatial proximity of these regions is not accounted for when performing a sliding window analysis, and it is only when using spatial information that we observe the highest Tajima’s D score of 2.39, compared to 1.84 for a linear sliding window calculation.

## Discussion

This study has examined structured protein domains from *P. falciparum* and the importance of structural features in assessing potential targets of humoral immunity. Antigenic polymorphism data from a Gambian population was used to identify regions under immune selection pressure, with polymorphic residues found to be primarily surface exposed and enriched within turns as compared to other secondary structure elements. A number of studies have observed that polymorphism variation in buried residues is generally more detrimental to protein function than polymorphic variation in surface exposed regions^76–78^. This observation, coupled with antibody-driven selection pressure on surface-exposed residues, likely explains our finding that high-frequency polymorphic residues were highly surface exposed. Similarly, our observation that polymorphic residues were proportionally enriched within turns is supported by previous work examining the structural features of antigen-antibody interfaces^79–81^. These studies used a three-state definition of secondary structure (helices, sheets, loops), and observed an increased proportion of loop elements (turns, bends or coils) within epitopes; we only observed an increased number of polymorphisms within turns.

Prediction of B-cell epitopes is a challenging problem, with early attempts based on amino acid residue propensity scales only a little better than chance^82^. Despite many advances in recent years, the performance of current B-cell epitope prediction algorithms still falls behind that of predictors of other immunological features, such as MHC and T-cell epitope predictors^83^. With this in mind, the results obtained in our study are encouraging. The sequence-based BepiPred 2.0 algorithm^41^ predominantly identifies surface exposed regions as potential epitopes, despite only using the linear protein sequence as an input. Additionally, when restricting our analysis to only surface exposed residues, ~75% of polymorphic residues were placed within predicted epitopes compared to ~50% of non-polymorphic residues (at the default threshold). Thus, we suggest that BepiPred 2.0 is an informative tool for initial selection of potential epitopes, particularly in the absence of a known protein structure.

Another approach to identifying potential epitopes explored in this study was the identification of polymorphisms clustered within a potential antibody binding radius. All of the proteins identified using this approach are known highly polymorphic antigens, including AMA1, PfEMP1, EBA-175, TRAP, DBLMSP2, CSP and MSP1^84–89^. Additionally, our relatively conservative homology modelling approach did not yield additional proteins of interest when examining polymorphic hotspots, despite a 3-fold increase in the coverage of the proteome. Future work could extend the approach used in this study using structural models derived from other methods such as threading and fold recognition.

Other methods for determining likely immune targets within a pathogen include measures of balancing selection such as Tajima’s D. Typically, Tajima’s D is applied as either a single metric over a whole gene, or as a sliding window over the genome. A number of studies of malaria antigens have also applied Tajima’s D as a sliding window over particular genes to identify regions of the protein under immune-mediated selection pressure^9,10,23–26^. However, for antibody-mediated selection pressure, a conformational epitope may contain residues that are distant in the linear protein sequence. Thus, we hypothesized that incorporation of structural information into a sliding window calculation of Tajima’s D may improve detection of regions under immune selection pressure. We developed a Python tool (BioStructMap) for spatial averaging, and applied it to both *Pf*AMA1 and EBA-175. While application of a spatially derived Tajima’s D to EBA-175 did not yield any major differences compared to a standard linear sliding window method, we revealed a region in *Pf*AMA1 bordering DII and DIII with a high spatially derived Tajima’s D value that was not observed using a traditional sliding window (Fig. 6). Interestingly, this region is composed of four distinct segments of protein that combine to form a surface-exposed face, which explains why a linear sliding window method failed to identify this discontinuous epitope (Fig. 7).

Previous studies also suggest that DIII may be an additional target of humoral immune responses. In a comparison of Tajima’s D between *Pf*AMA1 and *Pv*AMA1, the highest Tajima’s D values for *Pf*AMA1 were observed in DIII, in contrast to DI for *Pv*AMA1^10^, suggesting that DIII may play a significant role as a target for protective immune responses against *P. falciparum*. It has also been observed that a monoclonal antibody (1E10) against *Pf*AMA1 DIII acts synergistically with antibodies against other distant parts of the protein to inhibit merozoite growth, despite not having potent inhibitory capabilities on its own^67^. This suggests a potential role for antibodies targeting DIII in the context of a broader response against *Pf*AMA1, despite an anti-DIII response not being inhibitory in isolation. Population studies examining antibody levels against *Pf*AMA1 domains suggest that antibody responses against both DII and DIII are relatively rare^90^, however the recombinant protein constructs used in these studies fail to account for the considerable interaction between domains. Indeed, the region of *Pf*AMA1 with the strongest signature of balancing selection in our study was composed of residues from both DII and DIII, and antibodies targeting this epitope may not be identified in assays that use recombinant DII or DIII constructs. Taken together, these studies support our observation that a region bordering DII and DIII of *Pf*AMA1 may be an important target of humoral responses in the context of natural infection, as well as supporting the validity of our spatially derived Tajima’s D approach.

In this study, we have developed an approach to identify structured regions of *P. falciparum* proteins that are likely under some level of immune selection pressure, showing that polymorphic sites are predominantly surface exposed and enriched within turns. We applied a spatially derived Tajima’s D calculation to key antigens, identifying a region of *Pf*AMA1 between DII and DIII that was under a high degree of balancing selection. These methods and accompanying results have utility in the identification of proteins under balancing selection, furthering our understanding of functional immune targets during malaria infection. These approaches are also broadly applicable to other pathogens.

## Electronic supplementary material

Supplementary Information
