# Supplementary material for: Proteome-wide mapping of immune features onto Plasmodium protein three-dimensional structures
Source: Sci Rep. 2018 Mar 12;8:4355. doi: 10.1038/s41598-018-22592-3 (PMC5847524; doi:10.1038/s41598-018-22592-3)
Supplement: Supplementary file 1 — Supplementary Information [file 41598_2018_22592_MOESM1_ESM.pdf]

SUPPLEMENTARY INFORMATION

Proteome-wide mapping of immune features onto *Plasmodium* protein three-dimensional structures

Andrew J Guy, Vashti Irani, James G Beeson, Benjamin Webb, Andrej Sali, Jack S Richards, Paul A Ramsland

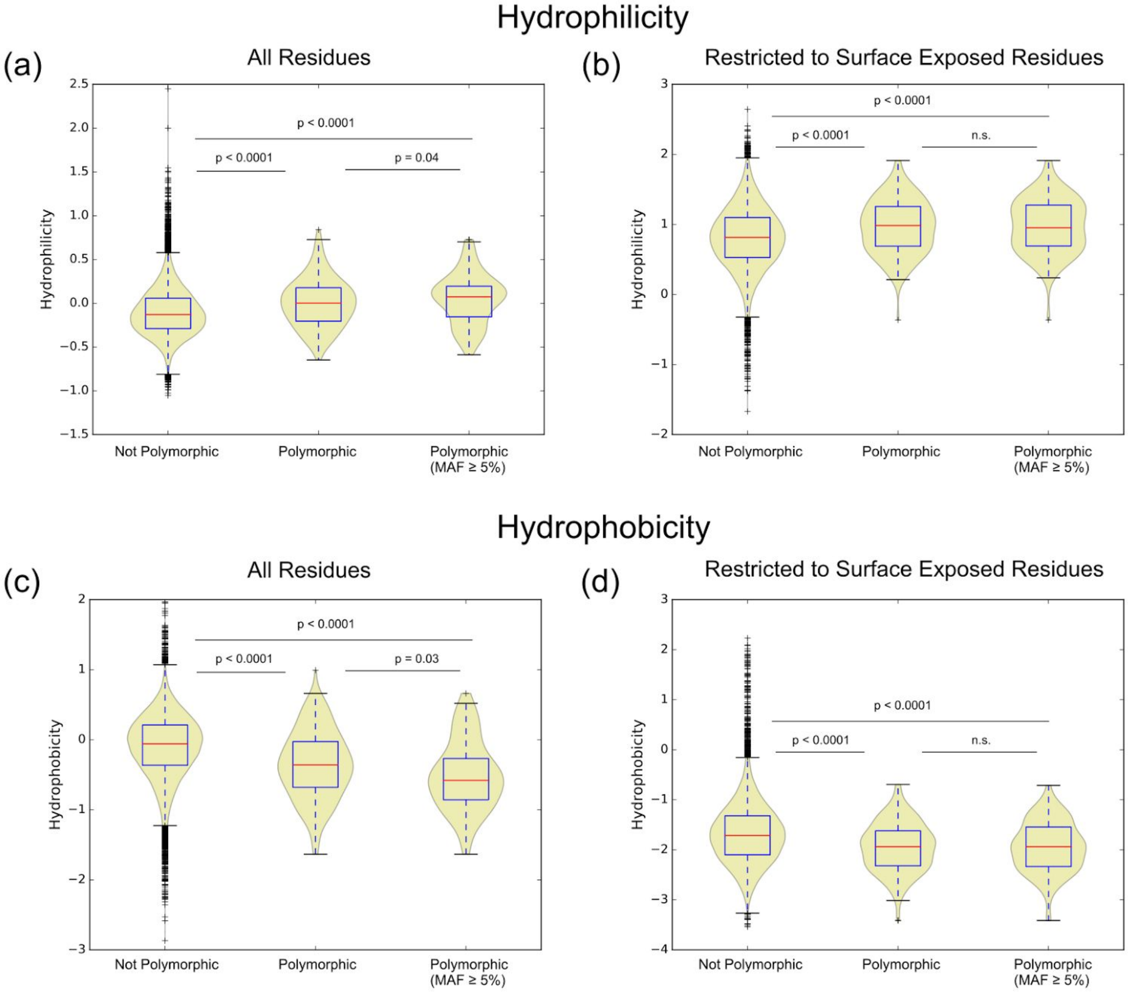

Figure S1: Average hydrophilicity and hydrophobicity are only weakly associated with residue polymorphisms once surface exposure is taken into account. A radius of 15 Å was used for spatial averaging of hydrophilicity and hydrophobicity values, and polymorphic residues identified by the presence of underlying non-synonymous SNPs, with additional filter using a minor allele frequency threshold of 5% also applied. **a)** Average hydrophilicity calculated over a 15 Å radius, grouped by residue polymorphism. **b)** Average hydrophilicity calculated over a 15 Å radius, with calculations restricted to surface exposed residues (RSA > 0.2) and grouped by residue polymorphism. **c)** Average hydrophobicity calculated over a 15 Å radius, grouped by residue polymorphism. **d)** Average hydrophobicity calculated over a 15 Å radius, with calculations restricted to surface exposed residues (RSA > 0.2) and grouped by residue polymorphism. Mann-Whitney U test used for comparison between groups.

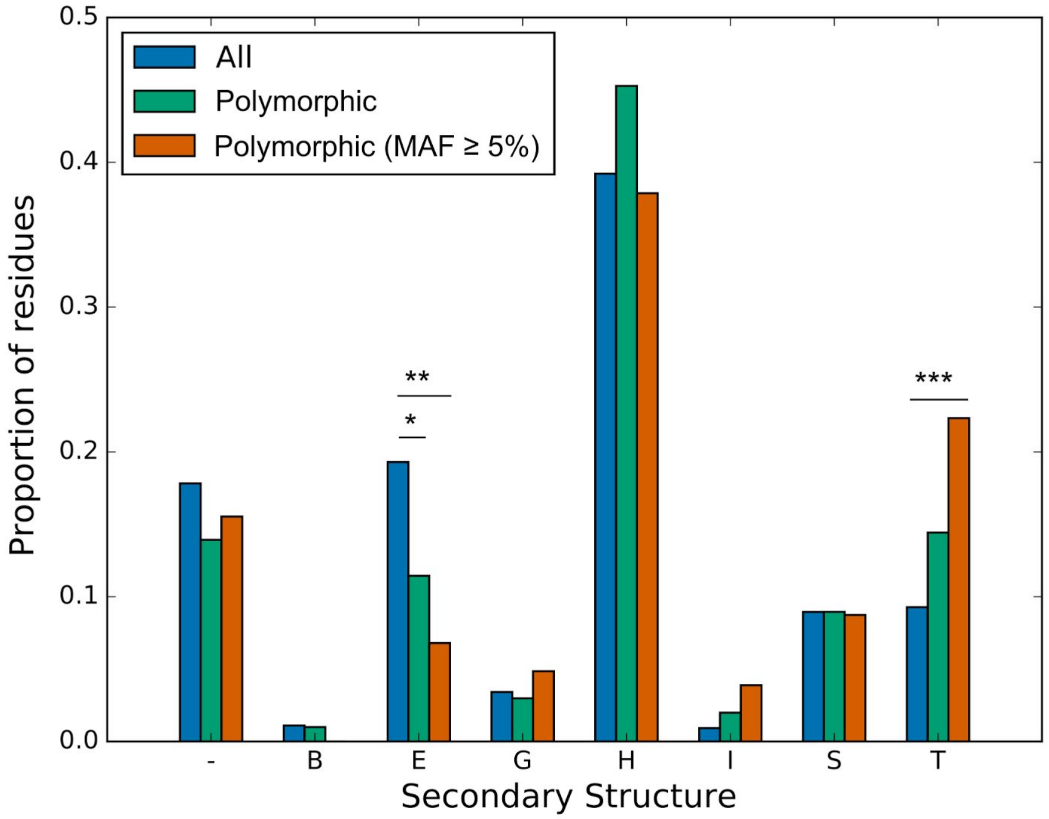

Figure S2: Polymorphic residues are more common within secondary structure turn elements and reduced within β-strand elements. Secondary structure elements for polymorphic residues were compared to the background set of protein structures from *P. falciparum*. When considering all polymorphic residues (without a MAF cutoff), a decreased proportion of polymorphic residues within β-strand (E) elements was observed ($p = 0.03$ after Bonferroni correction for multiple comparison; binomial test). When restricting polymorphic residues to those with a minor allele frequency (MAF) ≥ 5%, an increased proportion of polymorphic residues were contained in turn (T) elements ($p = 0.005$ after Bonferroni correction for multiple comparison; binomial test), and a reduced number of polymorphic residues found within within β-strand (E) elements ($p < 0.001$ after Bonferroni correction for multiple comparison; binomial test). Polymorphic residues with underlying non-synonymous SNPs with a minor allele frequency (MAF) ≥ 5% are shown in orange, whereas all polymorphic residues (no MAF threshold) are shown in green. Secondary structure was classified using the DSSP program. Secondary structure assignments are coded as follows: H, Alpha helix; B, Beta bridge; E, Strand; G, 3-Turn Helix; I, 5-Turn Helix; T, Turn; S, Bend; -, Other/Coil.

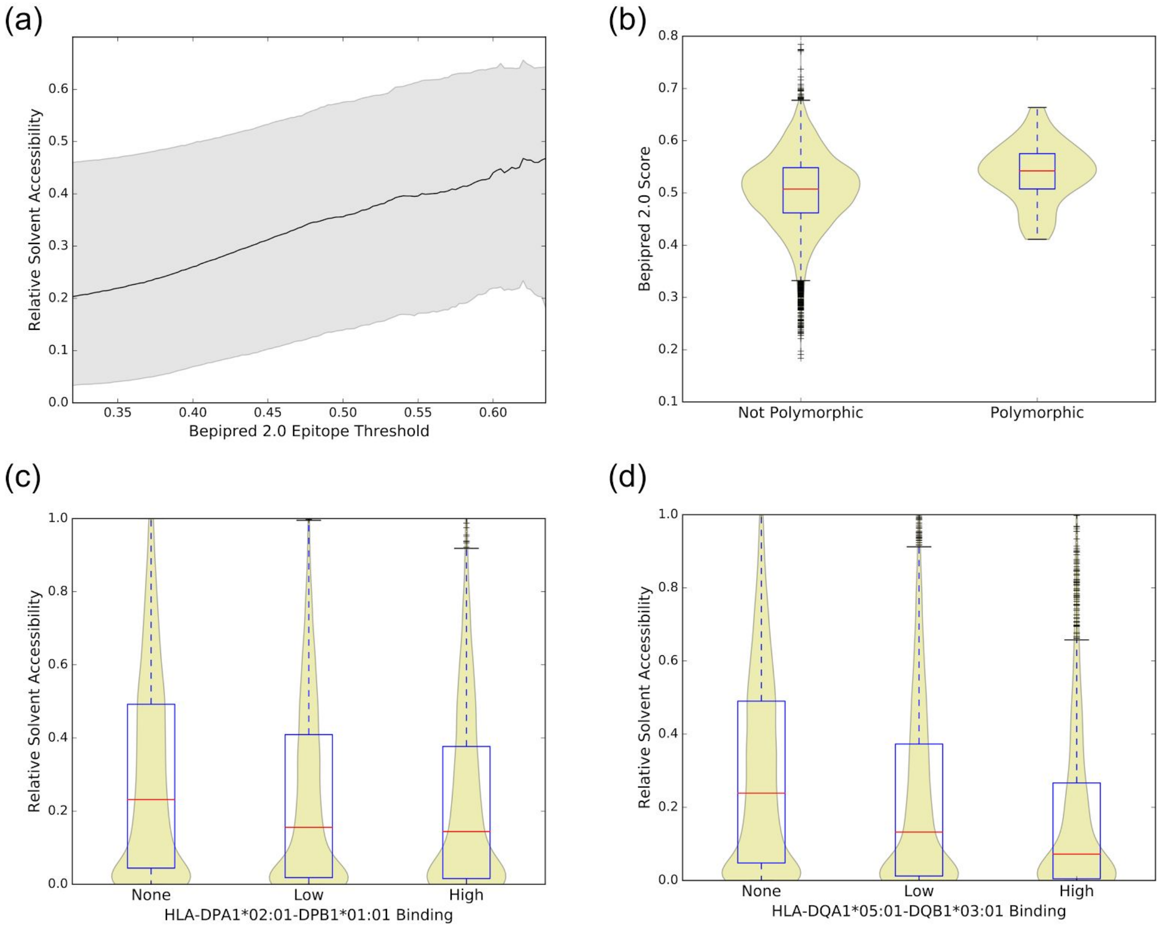

Figure S3: Predicted B-cell epitopes by Bepipred 2.0 strongly favor surface exposed residues, while predicted MHC II binding peptide are generally not surface exposed. a) Median relative solvent accessibility values were plotted for residues with an epitope score above any given threshold score for Bepipred 2.0. The location of the upper and lower quartiles are also shown in grey. We have shown results for thresholds between the 5th and 95th percentile of all scores for Bepipred 2.0. b) The distribution of Bepipred 2.0 scores was compared between polymorphic and non-polymorphic (MAF $\geq$ 5%) residues, with analysis restricted to surface exposed residues (relative solvent accessibility $\geq$ 0.2). c, d) The location of MHC II binding peptides were assessed in relation to surface exposure, with the majority of residues involved in an MHC II binding peptide having relatively little surface exposure. Haplotypes HLA-DPA1*02:01-DPB1*01:01 (c) and HLA-DQA1*05:01-DQB1*03:01 (d) were assessed here, as these haplotypes have been shown to be common within a Gambian population and are also available for prediction using the NetMHCII tool. Prediction of peptide binding was performed using NetMHCII 2.2, with a 15 aa peptide length and default settings.

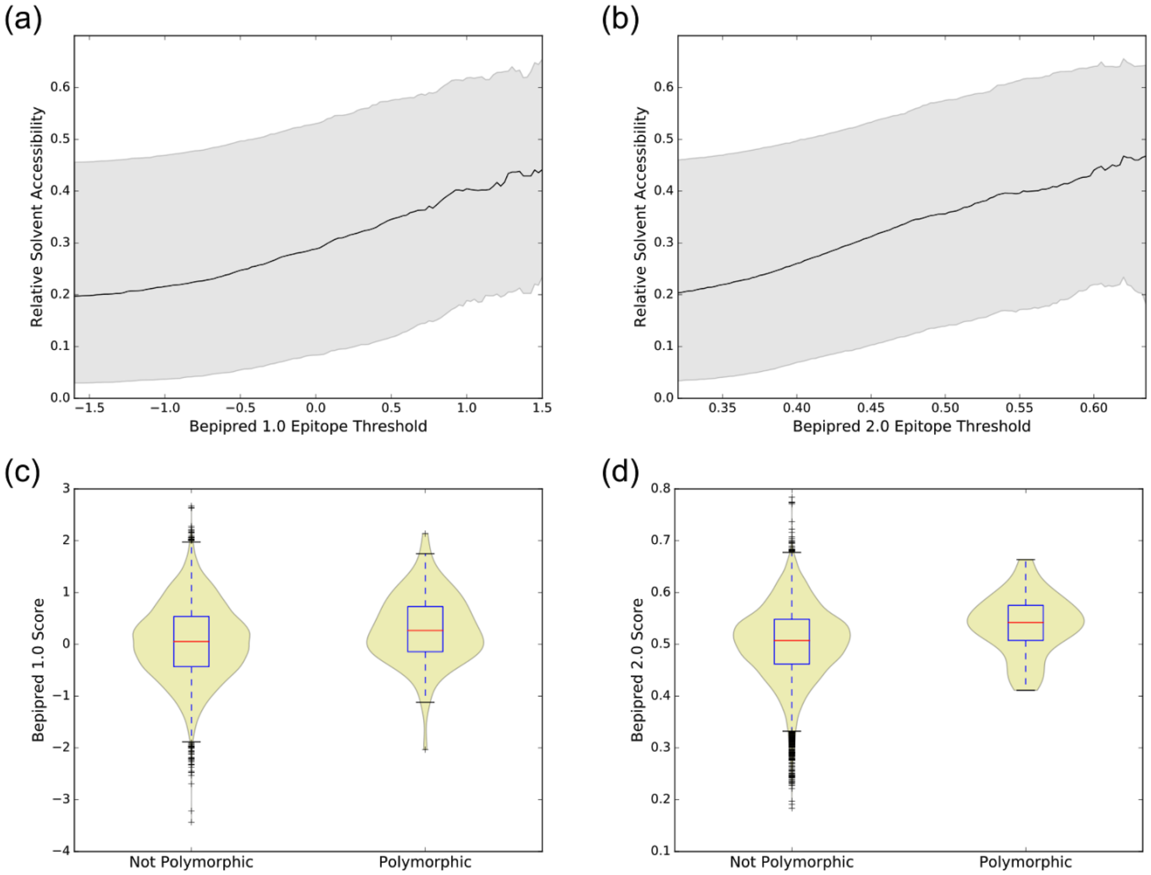

Figure S4: Predicted B-cell epitopes by both Bepipred 1.0 and Bepipred 2.0 both strongly favour surface exposed residues. Bepipred 2.0 performs better than Bepipred 1.0 in assigning polymorphic residues a high epitope probability. a, b) Median relative solvent accessibility values were plotted for residues with an epitope score above any given threshold score for Bepipred 1.0 **(a)** and Bepipred 2.0 **(b)**. The location of the upper and lower quartiles are also shown in grey. Note that the epitope scores used by Bepipred 1.0 and Bepipred 2.0 are not directly relatable, and hence we have shown results for thresholds between the 5th and 95th percentile of all scores for each predictor. Both Bepipred 1.0 and Bepipred 2.0 predicted epitopes were predominantly surface exposed, with increasing Bepipred thresholds predicting increasingly surface exposed residues. **c, d)** The distribution of Bepipred 1.0 **(c)** and Bepipred 2.0 **(d)** scores were compared between polymorphic and non-polymorphic (MAF $\geq$ 5%) residues, with analysis restricted to surface exposed residues (relative solvent accessibility ≥ 0.2). When considering the epitopes scores given to polymorphic residues, both Bepipred 1.0 and 2.0 scores were significantly higher for polymorphic residues (Bepipred 1.0, $p = 0.007$; Bepipred 2.0, $p < 0.0001$; Mann-Whitney U test), even when restricting analysis to surface exposed residues (relative solvent accessibility ≥ 0.2). It is noted that Bepipred 2.0 scores for polymorphic residues had the greater shift relative to the background distribution of epitope scores, with the median Bepipred 2.0 score for polymorphic residues shifted higher by 0.40 of the interquartile range (IQR) of the background distribution, compared to 0.22 of the IQR for Bepipred 1.0.

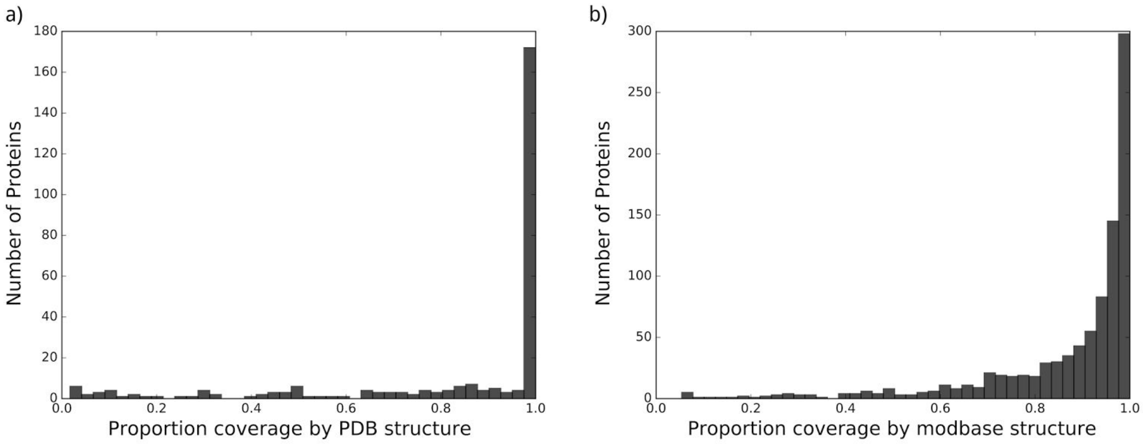

Figure S5: Most *P. falciparum* PDB structures and Modpipe models cover the majority of the reference protein sequence. (a) Proportion of *P. falciparum* reference protein sequence that is covered by a matching PDB structure (sequence similarity > 90%). A total of 275 *P. falciparum* proteins have at least one matching PDB structure. Proteins with no matching structure are not represented in this histogram. **(b)** Proportion of *P. falciparum* reference protein sequence that is covered by a high-quality ModPipe model (MPQS > 1.1). A total of 923 *P. falciparum* proteins have at least one high-quality ModPipe model. Proteins with no high-quality ModPipe model are not represented in this histogram.

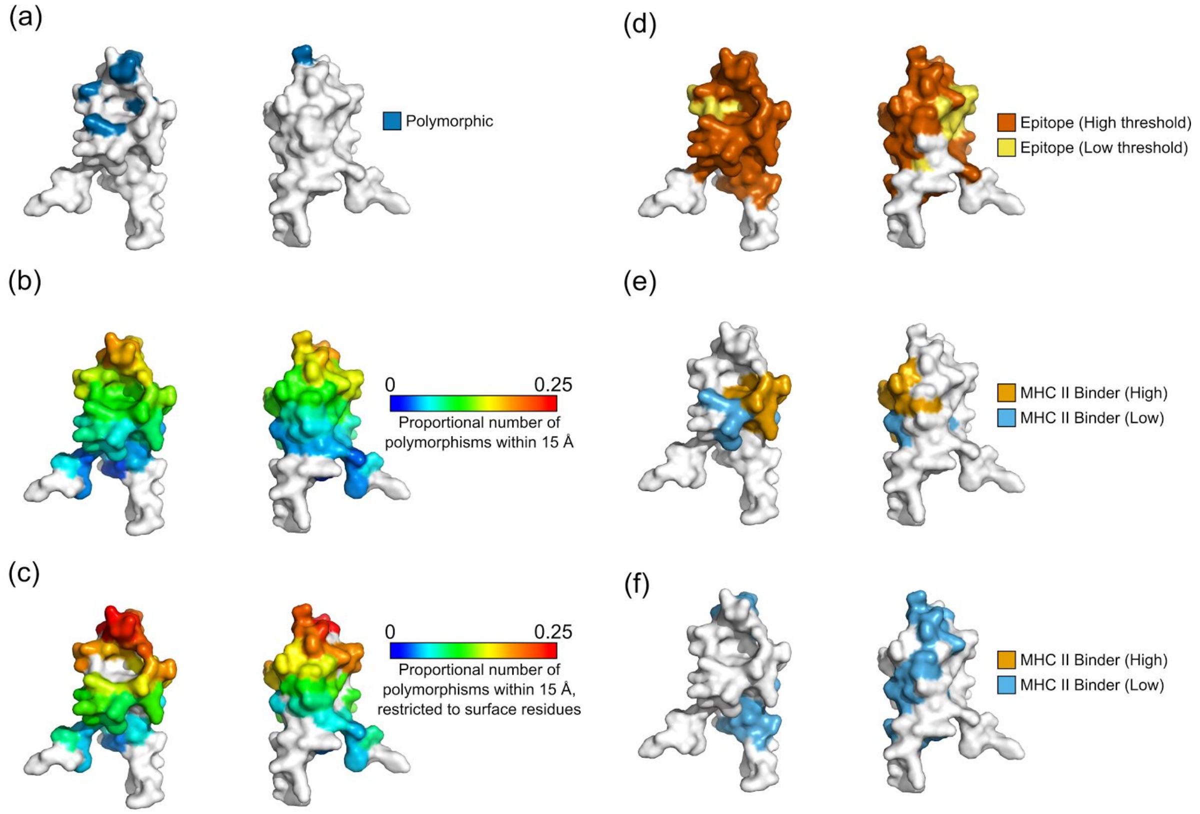

Figure S6: Location of immunologically relevant features mapped onto a CSP structure (PDB code: 3VDJ). Each panel shows the front and back and top view of the CSP structure. **a)** Polymorphic residues with an underlying minor allele frequency (MAF) greater than 5% are shown in blue. Sequence polymorphisms were obtained from 65 Gambian isolates[1]. **b)** Spatial averaging of polymorphic residues highlights polymorphic hotspots. The proportion of polymorphic residues within 15 Å is shown for each central residue, with polymorphic residues defined as those with a MAF ≥ 5% . **c)** Spatial averaging of polymorphic residues, restricted to surface exposed residues. The proportion of polymorphic residues within 15 Å is shown for each central residue, with polymorphic residues defined as those with a MAF ≥ 5% and surface exposed residues considered to be those with RSA ≥ 0.2. **d)** Bepipred 2.0 predictions, with epitopes shown for two Bepipred thresholds — predicted epitopes are shown in yellow for a threshold of 0.5 (specificity = 0.57, sensitivity = 0.59) and in dark orange for a threshold of 0.55 (specificity = 0.81, sensitivity = 0.29). **e, f)** The location of predicted MHC class II binding peptides are shown for the HLA-DPA1*02:01-DPB1*01:01 **(e)** and HLA-DQA1*05:01-DQB1*03:01 **(f)** alleles. Residues involved in a low binding peptide (50 nM < IC50 < 500 nM) are shown in light blue, while residues involved in a high binding peptide (IC50 < 50 nM) residue are shown in orange. Only the core binding region of each peptide binder is indicated on each structure.

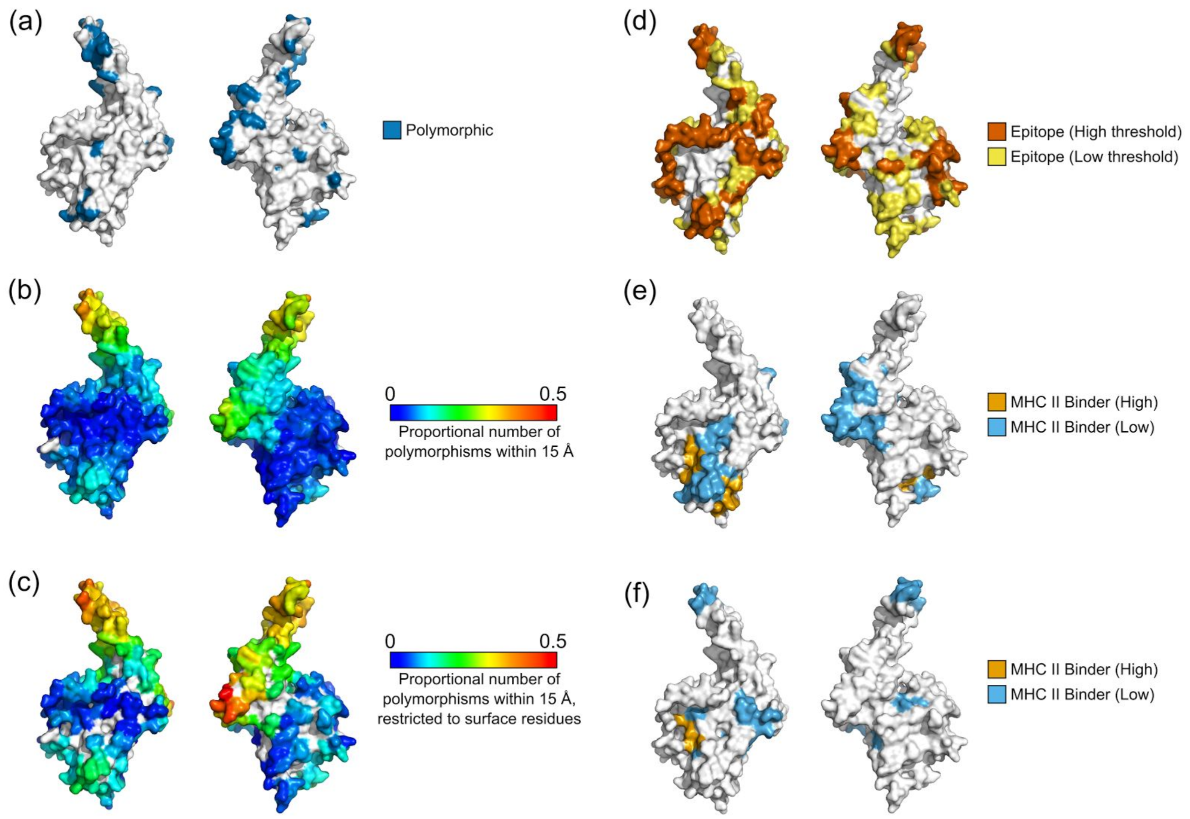

Figure S7: Location of immunologically relevant features mapped onto an MSPDBL2 structural model *(ModPipe ID: 45d80d0181671431333fb05b6011a7e4)***.** Each panel shows the front and back and top view of the MSPDBL2 structure. **a)** Polymorphic residues with an underlying minor allele frequency (MAF) greater than 5% are shown in blue. Sequence polymorphisms were obtained from 65 Gambian isolates[1]. **b)** Spatial averaging of polymorphic residues highlights polymorphic hotspots. The proportion of polymorphic residues within 15 Å is shown for each central residue, with polymorphic residues defined as those with a MAF ≥ 5% . **c)** Spatial averaging of polymorphic residues, restricted to surface exposed residues. The proportion of polymorphic residues within 15 Å is shown for each central residue, with polymorphic residues defined as those with a MAF ≥ 5% and surface exposed residues considered to be those with RSA ≥ 0.2. **d)** Bepipred 2.0 predictions, with epitopes shown for two Bepipred thresholds — predicted epitopes are shown in yellow for a threshold of 0.5 (specificity = 0.57, sensitivity = 0.59) and in dark orange for a threshold of 0.55 (specificity = 0.81, sensitivity = 0.29). **e, f)** The location of predicted MHC class II binding peptides are shown for the HLA-DPA1*02:01-DPB1*01:01 **(e)** and HLA-DQA1*05:01-DQB1*03:01 **(f)** alleles. Residues involved in a low binding peptide (50 nM < IC50 < 500 nM) are shown in light blue, while residues involved in a high binding peptide (IC50 < 50 nM) residue are shown in orange. Only the core binding region of each peptide binder is indicated on each structure.

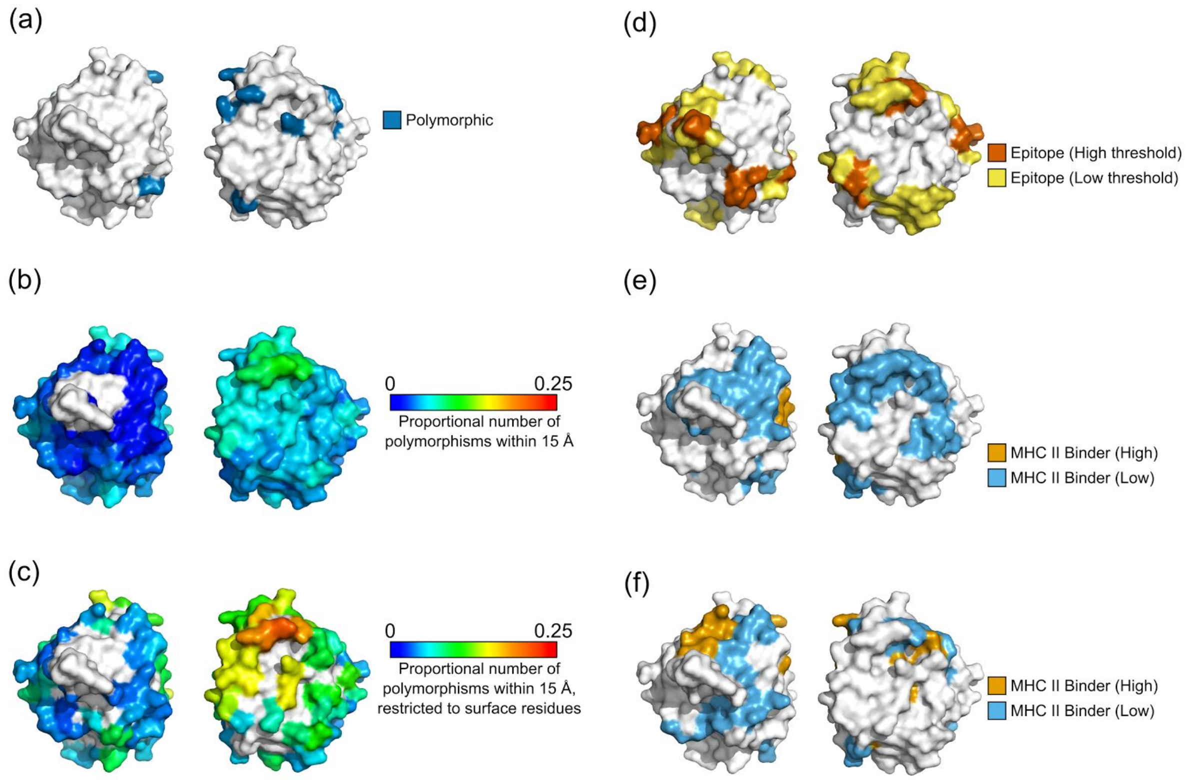

Figure S8: Location of immunologically relevant features mapped onto a TRAP structure (PDB code: 4F1J). Each panel shows the front and back and top view of the TRAP structure. **a)** Polymorphic residues with an underlying minor allele frequency (MAF) greater than 5% are shown in blue. Sequence polymorphisms were obtained from 65 Gambian isolates[1]. **b)** Spatial averaging of polymorphic residues highlights polymorphic hotspots. The proportion of polymorphic residues within 15 Å is shown for each central residue, with polymorphic residues defined as those with a MAF ≥ 5% . **c)** Spatial averaging of polymorphic residues, restricted to surface exposed residues. The proportion of polymorphic residues within 15 Å is shown for each central residue, with polymorphic residues defined as those with a MAF ≥ 5% and surface exposed residues considered to be those with RSA ≥ 0.2. **d)** Bepipred 2.0 predictions, with epitopes shown for two Bepipred thresholds — predicted epitopes are shown in yellow for a threshold of 0.5 (specificity = 0.57, sensitivity = 0.59) and in dark orange for a threshold of 0.55 (specificity = 0.81, sensitivity = 0.29). **e, f)** The location of predicted MHC class II binding peptides are shown for the HLA-DPA1*02:01-DPB1*01:01 **(e)** and HLA-DQA1*05:01-DQB1*03:01 **(f)** alleles. Residues involved in a low binding peptide (50 nM < IC50 < 500 nM) are shown in light blue, while residues involved in a high binding peptide (IC50 < 50 nM) residue are shown in orange. Only the core binding region of each peptide binder is indicated on each structure.

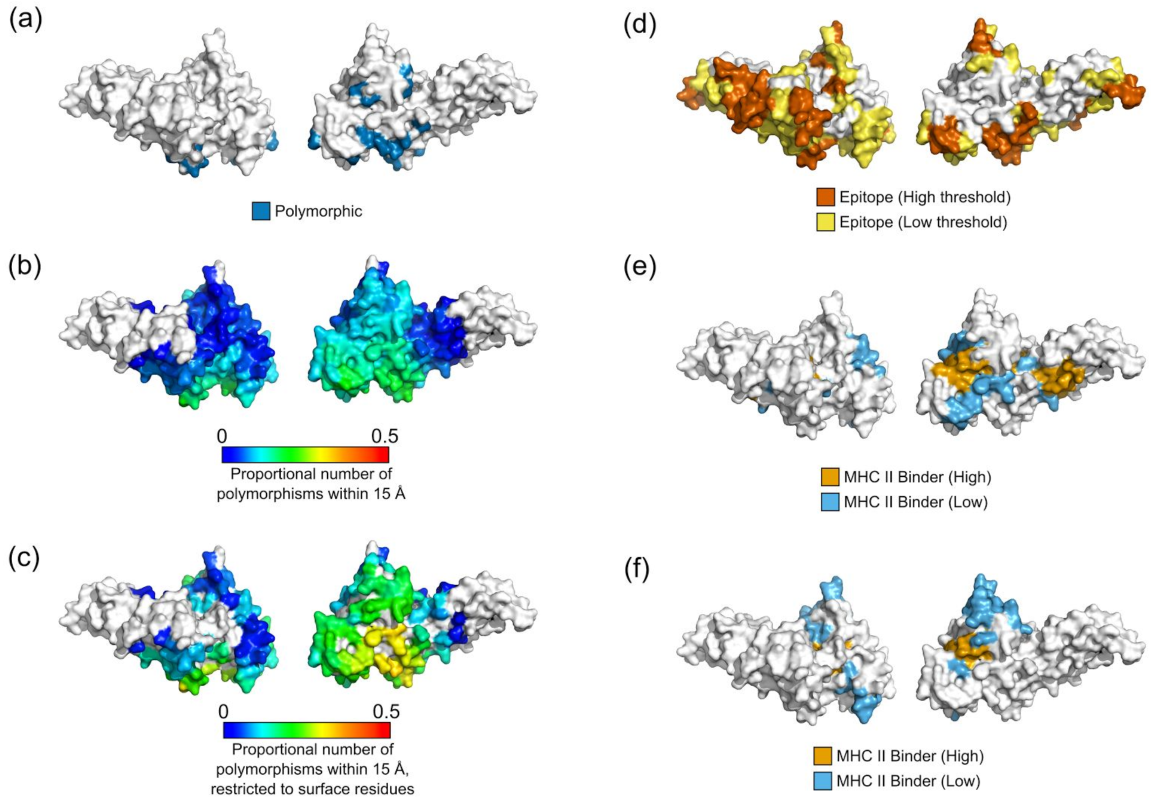

Figure S9: Location of immunologically relevant features mapped onto an PfEMP1 DBL6 structural model *(ModPipe ID: 5f1239b7c2f20f9a5455c9a43f0fa2d7)***.** Each panel shows the front and back and top view of the PfEMP1 DBL6 structure. **a)** Polymorphic residues with an underlying minor allele frequency (MAF) greater than 5% are shown in blue. Sequence polymorphisms were obtained from 65 Gambian isolates[1]. **b)** Spatial averaging of polymorphic residues highlights polymorphic hotspots. The proportion of polymorphic residues within 15 Å is shown for each central residue, with polymorphic residues defined as those with a MAF ≥ 5% . **c)** Spatial averaging of polymorphic residues, restricted to surface exposed residues. The proportion of polymorphic residues within 15 Å is shown for each central residue, with polymorphic residues defined as those with a MAF ≥ 5% and surface exposed residues considered to be those with RSA ≥ 0.2. **d)** Bepipred 2.0 predictions, with epitopes shown for two Bepipred thresholds — predicted epitopes are shown in yellow for a threshold of 0.5 (specificity = 0.57, sensitivity = 0.59) and in dark orange for a threshold of 0.55 (specificity = 0.81, sensitivity = 0.29). **e, f)** The location of predicted MHC class II binding peptides are shown for the HLA-DPA1*02:01-DPB1*01:01 **(e)** and HLA-DQA1*05:01-DQB1*03:01 **(f)** alleles. Residues involved in a low binding peptide (50 nM < IC50 < 500 nM) are shown in light blue, while residues involved in a high binding peptide (IC50 < 50 nM) residue are shown in orange. Only the core binding region of each peptide binder is indicated on each structure.

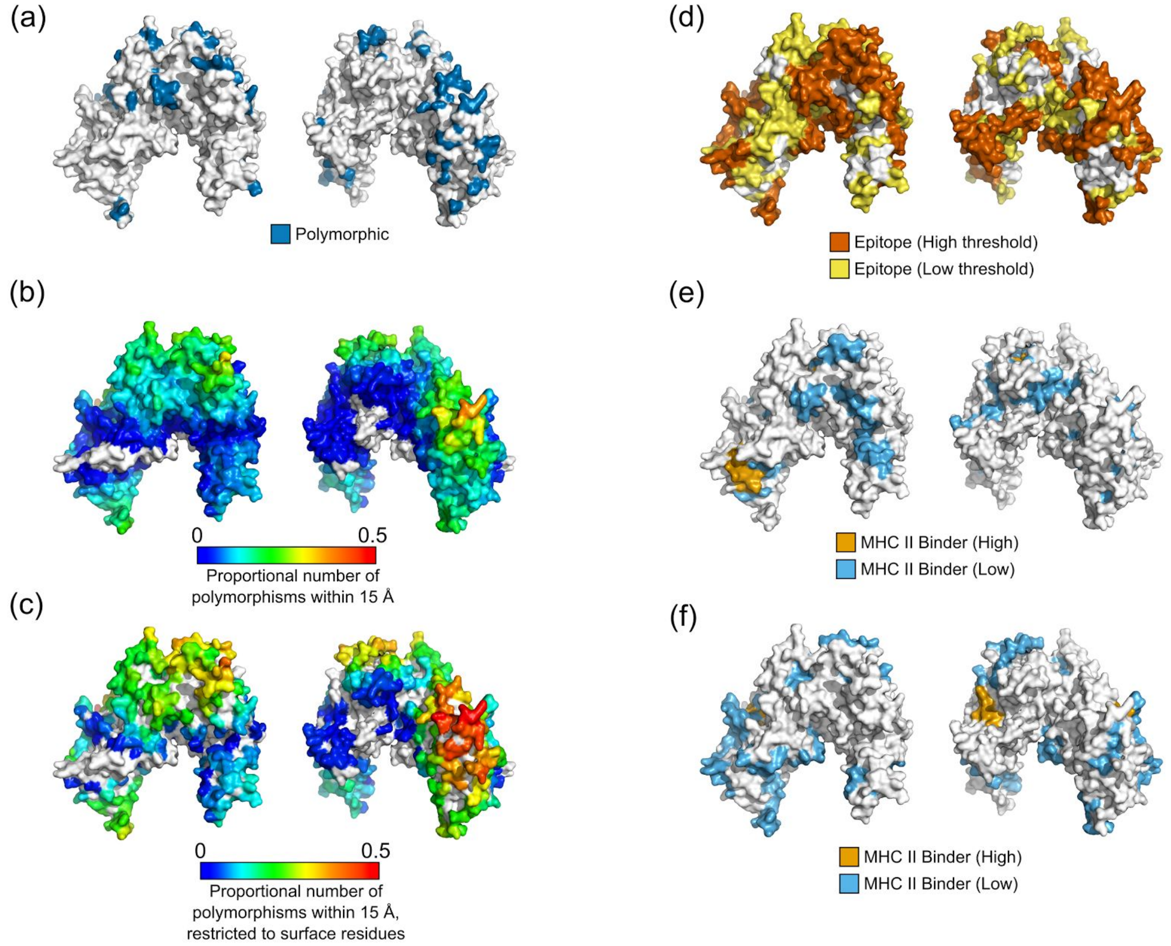

Figure S10: Location of immunologically relevant features mapped onto an PfEMP1 DBL3x-4e structural model *(ModPipe ID: 75fcc4663ff09a106096e5fd1e8ad13a)***.** Each panel shows the front and back and top view of the PfEMP1 DBL3x-4e structure. **a)** Polymorphic residues with an underlying minor allele frequency (MAF) greater than 5% are shown in blue. Sequence polymorphisms were obtained from 65 Gambian isolates[1]. **b)** Spatial averaging of polymorphic residues highlights polymorphic hotspots. The proportion of polymorphic residues within 15 Å is shown for each central residue, with polymorphic residues defined as those with a MAF ≥ 5% . **c)** Spatial averaging of polymorphic residues, restricted to surface exposed residues. The proportion of polymorphic residues within 15 Å is shown for each central residue, with polymorphic residues defined as those with a MAF ≥ 5% and surface exposed residues considered to be those with RSA ≥ 0.2. **d)** Bepipred 2.0 predictions, with epitopes shown for two Bepipred thresholds — predicted epitopes are shown in yellow for a threshold of 0.5 (specificity = 0.57, sensitivity = 0.59) and in dark orange for a threshold of 0.55 (specificity = 0.81, sensitivity = 0.29). **e, f)** The location of predicted MHC class II binding peptides are shown for the HLA-DPA1*02:01-DPB1*01:01 **(e)** and HLA-DQA1*05:01-DQB1*03:01 **(f)** alleles. Residues involved in a low binding peptide (50 nM < IC50 < 500 nM) are shown in light blue, while residues involved in a high binding peptide (IC50 < 50 nM) residue are shown in orange. Only the core binding region of each peptide binder is indicated on each structure.

Table S1: Polymorphic hotspots across all *P. falciparum* structures in the PDB, using data on polymorphisms from 65 Gambian isolates. Analysis was restricted to surface exposed residues (RSA > 0.2). A minor allele frequency threshold of 5% was used to identify immunologically relevant polymorphisms.

		**Location of residues with more than _% polymorphic residues within a 15Å radius**	
**Gene ID**	**Description**	**>10% polymorphic**	**>20% polymorphic**
PF3D7_1335900	thrombospondin-related anonymous protein (TRAP)	57, 60-63, 65-66, 73, 77, 94, 98-99, 104-105, 109, 112-113, 116-117, 119-129, 152	125
PF3D7_0731500	erythrocyte binding antigen-175 (EBA175)	399-400, 402-407, 481-482, 577-585, 588, 592, 595	-
PF3D7_1036300	merozoite surface protein (DBLMSP2)	162, 166-167, 169-173, 176, 178, 180, 197-198, 200, 216-218, 221-227, 229, 232, 236, 251-252, 255, 261, 287, 290-291, 294-296, 316, 332-333, 335, 339, 342, 344, 355-356, 359-360, 362-364, 366-367, 369-371, 373-374, 388-399, 401-402, 404-406, 408-409, 412-413, 416, 419-427, 429-430, 432, 435, 438-440, 442-443, 445, 447-449, 452-453, 456-457	344, 359, 364, 366-367, 369-371, 373-374, 388-399, 401, 404, 416, 419-427, 429-430, 432, 435, 438-440, 443, 445, 447-449, 452-453, 456-457
PF3D7_1133400	apical membrane antigen 1 (AMA1)	115-116, 121, 124, 148, 164, 167-168, 174-175, 184-189, 192, 194-197, 199-201, 203-207, 209-213, 216, 219, 223-224, 235, 242-246, 281-283, 285-287, 293, 295-297, 299-301, 304-305, 329-330, 332, 339-340, 343-344, 404-405, 407, 424, 435-436, 479-480,	187-189, 192, 194-197, 199-201, 203-207, 209-213, 216, 219, 223, 242-246, 282, 285-286, 296

		483-484, 486-487, 489, 498-504, 508, 510, 512	
PF3D7_0206800	merozoite surface protein 2 (MSP2)*	35-37, 41-43, 45-46	-
PF3D7_0304600	circumsporozoite (CS) protein (CSP)	312-314, 317, 321-322, 324-331, 345, 347-349, 351-359, 361-362, 365-366	325, 349, 352-357
PF3D7_1200600	erythrocyte membrane protein 1, PfEMP1 (VAR2CSA)	2350, 2357-2358, 2360-2362, 2364, 2373-2374, 2376, 2378, 2380-2382, 2385-2386, 2388-2393, 2395-2396, 2399-2400, 2403-2404, 2407-2408, 2410-2417, 2419, 2422, 2425, 2437-2438, 2442-2443, 2447-2448, 2454, 2457-2459, 2472, 2483-2484, 2487-2488, 2570-2571, 2574, 2578-2579	2357-2358, 2360-2361, 2373-2374, 2376, 2378, 2380-2382, 2385, 2393, 2404, 2407-2408, 2442-2443, 2447-2448
PF3D7_0930300	merozoite surface protein 1 (MSP1)	1614, 1639, 1669, 1671-1672, 1674-1676, 1679, 1681, 1687-1688	-
PF3D7_1115700	cysteine proteinase falcipain 2a	257	-
PF3D7_1115300	cysteine proteinase falcipain 2b	255	-
PF3D7_0906500	arginase	154	-

*The MSP2 crystal structure used here is a fragment of MSP2 in complex with an antibody Fv fragment (PDB code = 4QY8) and is not likely to be representative of the native structure of this protein—this region of MSP2 is thought to interact with the parasite plasma membrane [2].

Table S2: Polymorphic hotspots across all modelled *P. falciparum* structures with MPQS > 1.1, using data on polymorphisms from 65 Gambian isolates. Analysis was restricted to surface exposed residues (RSA > 0.2). A minor allele frequency threshold of 5% was used to identify immunologically relevant polymorphisms.

		**Location of residues with more than _% polymorphic residues within a 15Å radius**	
**Gene ID**	**Description**	**>10% polymorphic**	**>20% polymorphic**
PF3D7_0304600	circumsporozoite (CS) protein (CSP)	317-318, 321-322, 324-330, 345, 347-349, 351-359, 361-362, 365-366	349, 352-354, 356-357, 362
PF3D7_0731500	erythrocyte binding antigen-175 (EBA-175)	229, 329-330, 333, 336, 396, 399-400, 403-407, 409, 412, 577-584, 588, 592, 595	-
PF3D7_0930300	merozoite surface protein 1 (MSP1)	1671-1672, 1674, 1676	-
PF3D7_1335900	thrombospondin-related anonymous protein (TRAP)	39, 43, 46, 57, 60-62, 65-66, 73, 98-100, 104-106, 109, 112-113, 115-117, 119-120, 122-126, 128-129, 148, 152, 238-239, 290	125
PF3D7_1036300	duffy binding-like merozoite surface protein 2 (DBLMSP2)	166-167, 169-170, 172-173, 183, 197-198, 200, 216-218, 221-227, 229, 232, 252, 255, 287, 290-291, 294-296, 332-333, 339, 342, 344, 356, 359-360, 362-364, 366-370, 373-395, 397-399, 401-402, 405-406, 408-409, 412-413, 416, 419-427, 429-430, 432, 435, 438-440, 442, 445, 447-449, 452-453, 456-457	344, 359, 363-364, 366-370, 373-395, 397-399, 401, 416, 419-427, 429-430, 432, 435, 438-440, 445, 447-449, 452-453, 456
PF3D7_1133400	apical membrane antigen 1 (AMA1)	115-116, 118, 121, 124, 148, 164, 167-168, 171, 184-190, 192, 194, 196-197, 199-201, 203-207, 209-213, 219, 223-224, 235, 242-246, 267-268, 270, 281-283, 285-286, 293, 295-297, 299-301, 304-305, 332, 335, 339-340, 343-344, 404-405, 407-408, 423-424, 435-438, 440, 483-486, 489, 492-493,	187-190, 192, 194, 196-197, 199-201, 203-207, 209-213, 219, 223-224, 242-246, 282-283, 285-286, 436

		495-498, 501, 503, 508	
PF3D7_1200600	erythrocyte membrane protein 1, PfEMP1	1209-1215, 1217, 1219-1220, 1222-1223, 1231-1233, 1267, 1280-1281, 1283-1286, 1289, 1300, 1304, 1307-1308, 1315, 1317-1323, 1325-1327, 1329-1333, 1340, 1343, 1368-1369, 1372-1373, 1377-1379, 1381-1392, 1395-1396, 1399-1400, 1403-1404, 1407, 1410, 1414, 1417-1425, 1427-1429, 1432-1434, 1437-1438, 1461-1462, 1464-1465, 1467-1469, 1471-1478, 1480-1482, 1484-1486, 1488-1489, 1491-1492, 1495, 1502, 1505, 1524, 1526, 1534, 1539-1542, 1544-1546, 1548, 1557, 1559-1561, 1564-1572, 1574, 1579-1580, 1582, 1624, 1627-1631, 1633-1634, 1653, 1655, 1657, 1662-1663, 1718, 1722, 1725-1742, 1744, 1751, 1758, 1762-1771, 1773, 1776, 1779, 1809, 1817-1827, 1829-1830, 1837, 1872, 1911-1913, 1915-1916, 2350-2351, 2353-2355, 2357-2362, 2364, 2373-2374, 2376, 2378, 2380-2383, 2385-2386, 2388-2393, 2395-2397, 2400, 2403-2404, 2407, 2410-2417, 2419, 2422, 2425, 2437-2438, 2442-2443, 2447-2448, 2451, 2457-2459, 2480, 2483-2485, 2487-2488, 2521, 2525, 2567, 2570-2571, 2574, 2578-2579	1209-1215, 1217, 1284-1286, 1289, 1300, 1304, 1307-1308, 1317-1323, 1325-1327, 1329-1333, 1340, 1377-1379, 1381-1391, 1400, 1403-1404, 1407, 1410, 1414, 1417-1425, 1427-1429, 1432-1433, 1461, 1464, 1468, 1472, 1474-1477, 1484, 1488, 1526, 1539-1542, 1544-1546, 1548, 1559-1561, 1564-1566, 1568-1572, 1574, 1580, 1629-1631, 1634, 1653, 1726-1742, 1744, 1751, 1762-1771, 1773, 2353-2355, 2357-2362, 2373-2374, 2376, 2378, 2380-2383, 2393, 2397, 2407, 2411-2412, 2442-2443, 2447-2448
PF3D7_1115700	cysteine proteinase falcipain 2a	257	-
PF3D7_1115300	cysteine proteinase falcipain 2b	255	-
PF3D7_0519200	V-type proton ATPase 16 kDa proteolipid	5	-

	subunit		
PF3D7_0919500	major facilitator superfamily domain-containing protein, putative	236	-

SUPPLEMENTARY REFERENCES

1. Amambua-Ngwa, A. *et al.* Population Genomic Scan for Candidate Signatures of Balancing Selection to Guide Antigen Characterization in Malaria Parasites. *PLoS Genet.* 8, e1002992 (2012).
2. Adda, C. G. *et al.* Antigenic characterization of an intrinsically unstructured protein, Plasmodium falciparum merozoite surface protein 2. *Infect. Immun.* 80, 4177–4185 (2012).
